# Targeting the intracellular signaling “STOP” and “GO” pathways for the treatment of alcohol use disorders

**DOI:** 10.1007/s00213-018-4882-z

**Published:** 2018-04-14

**Authors:** Dorit Ron, Anthony Berger

**Affiliations:** Department of Neurology, University of California San Francisco, San Francisco, CA USA

**Keywords:** Alcohol, Addiction, Signaling, Translation, Medication Development, Fyn, mTOR, BDNF, GDNF

## Abstract

In recent years, research has identified the molecular and neural substrates underlying the transition of moderate “social” consumption of alcohol to the characteristic alcohol use disorder (AUD) phenotypes including excessive and compulsive alcohol use which we define in the review as the GO signaling pathways. In addition, growing evidence points to the existence of molecular mechanisms that keep alcohol consumption in check and that confer resilience for the development of AUD which we define herein as the STOP signaling pathways. In this review, we focus on examples of the GO and the STOP intracellular signaling pathways and discuss our current knowledge of how manipulations of these pathways may be used for the treatment of AUD.

## Introduction

Alcohol use disorder (AUD) is a serious worldwide health problem afflicting 15% of the population (Sridhar 2012; WHO 2014), causing significant medical, social, and economic burdens, in large part due to limited treatment options (Whiteford et al. 2013). Strikingly, the use of alcohol is widespread with over 70% of the USA population reporting drinking alcohol in the past 12 months (Grant et al. 2017). The need to understand the mechanisms that drive or prevent excessive drinking is underscored by a recent report indicating that the prevalence of AUD has increased by 35% in the USA between 2001 and 2013 (Grant et al. 2017). Despite an increase in alcohol use and abuse amongst all sociodemographic groups in the USA, some groups have significantly higher rates of problem drinking (Grant et al. 2017), indicating significant vulnerabilities amongst some individuals and a pressing need for the development of treatment options for these groups.

Unfortunately, pharmacotherapy options for adverse phenotypes such as binge drinking, craving, seeking, dependence, and relapse are rather limited (Wackernah et al. 2014), and medications such as naltrexone, acamprosate, and disulfiram not only are beneficial but also suffer from efficacy and compliance issues (Wackernah et al. 2014). Thus, there is a growing need to develop more efficacious and safer therapies to treat aspects of AUD. In addition, clinical trials are sorely needed to allow physicians to prescribe Food and Drug Administration (FDA)-approved medications which preclinical research, described herein, has identified as being potentially beneficial for the treatment of AUD.

The use of behavioral paradigms such as home cage alcohol drinking and operant self-administration enabled the investigation of mechanisms underlying phenotypes such as excessive consumption, seeking, dependence, and relapse (Carnicella et al. 2014; Lopez and Becker 2014; Vengeliene et al. 2014), and the use of these animal models has shed light on molecular signaling cascades that are recruited by alcohol. For example, and as described herein, the engagement of signaling components within corticostriatal circuitries following exposure to alcohol results in structural and functional changes (Ron and Barak 2016), which in turn influences an organism’s subsequent motivation to obtain alcohol (Koob and Volkow 2010).

This review will synthesize the current knowledge of molecular pathways in corticostriatal and amygdalar circuitries which drive the facilitation of alcohol drinking behaviors as well as the mechanisms that maintain alcohol intake in moderation. The focus on alcohol consumption over other phenotypes associated with AUD reflects the fact that voluntary consumption of alcohol subsequently influences maladaptive phenotypes such as alcohol seeking, craving, and relapse. Finally, this review will highlight promising therapeutic strategies for the treatment of AUD, and discuss future avenues of research critical for understanding the pathogenesis of AUD.

## STOP and GO signaling pathways

**Fig. 1:**
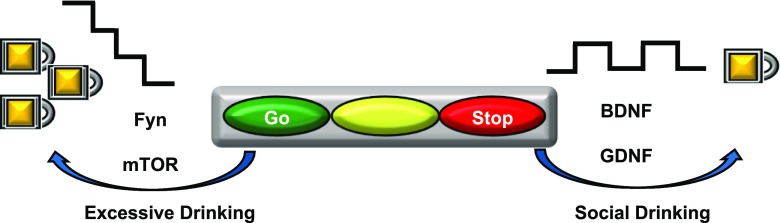
The STOP and GO intracellular signaling pathways control alcohol-drinking behaviors. Moderate and/or limited consumption of alcohol results in the activation of signaling cascades within the STOP pathways. STOP signaling pathways which center around genes such as BDNF and GDNF are described herein. Activation of the STOP signaling cascades limits the amount of consumed alcohol and keeps alcohol intake in check (*right panel*). Prolonged consumption of large quantities of alcohol activates the GO pathways. Activation of GO signaling cascades are driven in part, by the activation of Fyn and mTOR to produce neuroadaptations that contribute to the escalation and maintenance of excessive uncontrolled alcohol intake. The GO pathways are also the molecular underpinnings of other adverse phenotypes such as alcohol craving, seeking, and relapse (*left panel*)

**Fig. 2:**
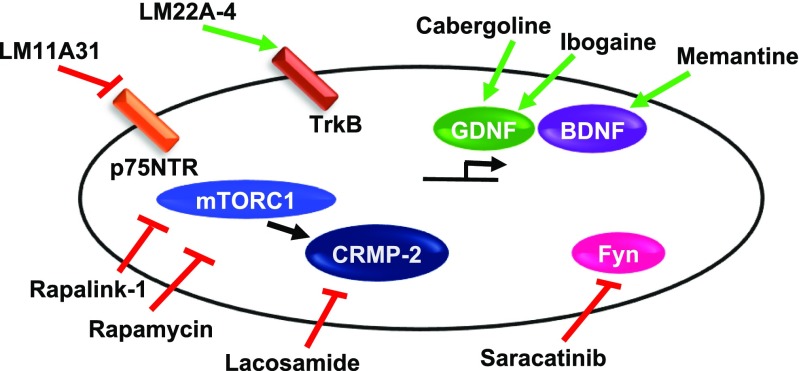
Drug candidates for the treatment of AUD. Preclinical animal models suggest that targeting GO and STOP signaling reduce alcohol-drinking behaviors as well as other behaviors associated with alcohol use. These include inhibitors of GO signaling and activators of STOP signaling. Specifically, cabergoline and ibogaine are inducers of GDNF expression. Memantine is an inducer of BDNF expression. LM22A-4 is an activator of the BDNF receptor TrkB, and LM11A31 is an inhibitor of the low affinity neurotrophin receptor, p75NTR. Saracatinib is a Fyn kinase inhibitor, and rapamycin and rapalink are mTORC1 inhibitors. Finally, lacosamide inhibits the function of CRMP2

We, and others, identified distinct molecular cascades that are recruited by alcohol consumption to either gate or promote the development of AUD. Specifically, we describe examples of signaling cascades termed herein as the STOP pathways which gate the development of excessive alcohol consumption and limit alcohol intake to moderate levels, and signaling cascades termed herein the GO pathways that drive the escalation of drinking from moderate to excessive levels and that contribute to the maintenance of high level of drinking, craving, and relapse (Ron and Barak 2016) (Fig. 1). This review does not discuss intracellular signaling components such as the adenylate cyclase/PKA/phosphodiesterase and protein kinase C (PKC) that also play an important role in phenotypes associated with AUD and have been reviewed elsewhere (Ahmadiantehrani et al. 2014b; Ron and Barak 2016). Finally, as outlined below, preclinical findings indicate that the manipulation of molecular components of the STOP and GO pathways could prove to be beneficial for the treatment of AUD (Fig. 2).

## STOP pathways moderate alcohol drinking behavior

Here, we focus on two genes, the brain-derived neurotrophic factor (BDNF) and the glial-derived neurotrophic factor (GDNF), and describe their role in gating alcohol use. We further show that malfunctioning of the BDNF and GDNF signaling pathways drives phenotypes associated with AUD. Finally, we present data to suggest that small molecules that target BDNF and GDNF signaling could be developed as medications to treat AUD (Fig. 2).

### BDNF

The neurotrophic factor BDNF is a member of the nerve growth factor (NGF) family (Huang and Reichardt 2003). BDNF binds to its high affinity receptor tropomyosin-related kinase B (TrkB) and with a low affinity to the p75 neurotrophin receptor (p75NTR) (Huang and Reichardt 2003; Kraemer et al. 2014b). Binding of BDNF with TrkB leads to the activation of the mitogen-activated protein kinase (MAPK)/extracellular signal-regulated kinases 1 and 2 (ERK1/2), phospholipase C gamma (PLC gamma), and phosphoinositol 3-kinase (PI3K) pathways (Huang and Reichardt 2003). Binding of mature or proBDNF to the p75NTR produces different outcomes including the phosphorylation of the c-Jun amino terminal kinase (JNK), the regulation of RhoA activity, the synthesis of ceramides, and the nuclear translocation of nuclear factor kappa-light-chain-enhancer of activated B cells (NFkB) (Kraemer et al. 2014b). The BDNF/TrkB pathway plays an important role in synaptic and structural plasticity and learning and memory (Bekinschtein et al. 2014; Panja and Bramham 2014; Park and Poo 2013), whereas p75NTR mediates opposite responses (Kraemer et al. 2014b). For example, activation of the p75NTR pathway reduces spine complexity and density (Zagrebelsky et al. 2005), induces apoptosis (Kraemer et al. 2014a) and long-term depression (LTD) (Woo et al. 2005).

Dysregulation of BDNF function has been implicated in multiple neuropsychiatric disorders (Autry and Monteggia 2012; Castren 2004), including depression (Duman and Li 2012), schizophrenia (Buckley et al. 2007), and anxiety disorders (Andero et al. 2014). Furthermore, the rapid actions of the anti-depressant medication, ketamine, are mediated in part by BDNF (Bjorkholm and Monteggia 2016).

### BDNF and alcohol

Initial studies elucidating the potential role of the growth factor in phenotypes associated with AUD utilized heterozygote *Bdnf* knockout animals which express approximately 50% of the *Bdnf* gene. Interestingly, *Bdnf* heterozygous mice (HET) consume more alcohol as compared to wild-type littermates (WT) (Hensler et al. 2003; McGough et al. 2004). Furthermore, deletion of *Bdnf* in the forebrain increases the consumption of a sweetened solution of alcohol without changing the level of saccharine intake (Logrip et al. 2015). *Bdnf* HET mice also display an enhanced alcohol conditioned place preference (CPP) as compared to their corresponding WT littermates (McGough et al. 2004), implicating the endogenous growth factor in gating alcohol intake and reward.

Further work indicates that *Bdnf* is an alcohol responsive gene. Specifically, moderate alcohol consumption (continuous access to 10% alcohol in a two bottle choice procedure; 10%CA-2BC), but not excessive intake (intermittent access to 20% alcohol; 20%IA-2BC), increases the levels of *BDNF* expression in the dorsal striatum of mice (McGough et al. 2004), and more specifically in the dorsolateral striatum (DLS), but not in the dorsomedial striatum (DMS) or nucleus accumbens (NAc) of rodents (Jeanblanc et al. 2009; Logrip et al. 2009b). Finally, in line with the negative correlation of BDNF with the level of alcohol consumed or the length of alcohol exposure, alcohol-preferring P rats (Li et al. 1987) exhibit lower BDNF content in the medial (MeA) and central (CeA) subregions of the amygdala as compared to non-preferring (NP) rats (Pandey et al. 2008; Yan et al. 2005). Whether BDNF levels differ in P and NP rats in corticostriatal regions regions has yet to be determined.

Experimental activation of TrkB signaling via infusion of recombinant BDNF in the DLS reduces operant alcohol self-administration (Jeanblanc et al. 2009), and the induction of *BDNF* expression through the systemic or intra-DLS administration of Tat-RACK1 (a recombinant RACK1 protein covertly linked to an amino acid sequence from the HIV-TAT protein that enables the penetration of proteins through the blood brain barrier and cellular membranes (Schwarze et al. 1999) reduces rat alcohol intake (Jeanblanc et al. 2006; McGough et al. 2004). However, infusion of BDNF in the DLS fails to alter rat operant self-administration of sucrose (Jeanblanc et al. 2009), indicating that BDNF’s actions are specific for alcohol. Conversely, RNA interference (RNAi) mediated knockdown of BDNF in the rat DLS but not DMS of increases alcohol self-administration (Jeanblanc et al. 2009), and similar data were obtain upon knockdown of BDNF in the DLS of mice (Logrip et al. 2015).

BDNF gates alcohol intake through the activation of ERK1/2, but not PI3K or PLC gamma (Jeanblanc et al. 2013). In addition, BDNF-mediated gating of alcohol intake requires a transcription/translation event that occurs within a time frame of hours (Jeanblanc et al. 2013). Specifically, activation of BDNF signaling initiates the transcription of the *dopamine D3 receptor* and *preprodynorphin*, the precursor to the dynorphin peptide in the dorsal striatum and in primary striatal neurons, respectively (Jeanblanc et al. 2006; Logrip et al. 2008). Finally, blockade of the dopamine D3 receptor (D3R) or the kappa opioid receptor (KOR), the receptor for dynorphin, reduces the ability of Tat-RACK1 to decrease alcohol consumption (Jeanblanc et al. 2006; Logrip et al. 2008). KOR and D3R are distributed in select brain regions involved in motivated behaviors (Bazov et al. 2018; Khaled et al. 2014), and upon activation, both serve to modulate dopaminergic neurotransmission (Bazov et al. 2018; Chang et al. 2018). Thus, it is plausible that the dynorphin/KOR system and the D3R in the DLS limit alcohol intake through their interaction with the dopaminergic mesolimbic system. Support for this possibility stems from the finding that systemic administration of the kappa opioid receptor agonist, U50,488H attenuates the rewarding properties of alcohol (Logrip et al. 2009a), and that the dopamine D3 receptor antagonist, U-99194A, increases alcohol-dependent place preference (Boyce and Risinger 2000, 2002).

Numerous lines of investigation in rodents have led to the interesting hypothesis that AUD occurs in part because of a breakdown of endogenous protective STOP signals. A breakdown in these signaling pathways can be the result of changes in the levels of the gene factor and/or the disruption of downstream signaling cascades. Studies have indicated that, at least in the case of BDNF, both possibilities are true. Specifically, long-term excessive consumption of alcohol disrupts the increase in BDNF expression in the DLS seen after the intake of moderate drinking of alcohol (Logrip et al. 2009b). The breakdown of BDNF signaling in the DLS coincides with a significant decrease of *BDNF* mRNA in cortical regions including the medial prefrontal cortex (mPFC) of mice and rats (Darcq et al. 2015; Logrip et al. 2009b; Smith et al. 2016; Tapocik et al. 2014). Furthermore, recent studies indicate that cortical BDNF is reduced following both chronic self-administration (Orru et al. 2016) and forced chronic intermittent exposure to alcohol (Fernandez et al. 2017). Interestingly, the decrease in *BDNF* expression in response to voluntary or non-contingent exposure of rodents to high levels of alcohol is mediated through the microRNA (miR) machinery (Heilig et al. 2017). miRs are short, noncoding RNA sequences that block mRNA translation by binding to cytoplasmic mRNA, targeting them for degradation (Bartel 2004), and alcohol increases the levels of miR30a-5p (Darcq et al. 2015) and miR206 (Tapocik et al. 2014) both of which target *BDNF* mRNA for degradation (Darcq et al. 2015; Tapocik et al. 2014). Importantly, inhibition of the function of miR30a-5p or miR206 inhibits excessive alcohol intake and prevents the escalation of alcohol drinking (Darcq et al. 2015; Tapocik et al. 2014). Interestingly, the increase of *BDNF* levels in the DLS during the consumption of moderate levels of alcohol could be also linked to miR function as the consumption of low level of alcohol led to a decrease in the expression of another *BDNF*-targeting miR, miR124a, in the DLS, thereby increasing BDNF mRNA levels (Bahi and Dreyer 2013).

Another potential neuroadaptation in response to excessive alcohol use is the breakdown of BDNF signaling. In fact, repeated cycles of alcohol drinking and withdrawal disrupt the balance of BDNF receptors by the forward trafficking of the low affinity BDNF receptor, p75NTR (Darcq et al. 2016). In support of the potential contribution of p75NTR to the escalation of alcohol use are the findings that knockdown of the p75NTR in the DLS or the pharmacological inhibition of the receptor reverts alcohol drinking to a moderate level (Darcq et al. 2016). Furthermore, an interesting possibility is that innate differences in BDNF levels and/or function are predisposing factors that determine whether or not subjects will escalate their consumption or drink alcohol in moderation. Interestingly, P rats consume more alcohol, exhibit heightened anxiety and have lower levels of BDNF in the CeA and MeA as compared to NP rats (Moonat et al. 2011; Moonat et al. 2013).

### BDNF in humans

The current human data broadly agrees with the role of BDNF in rodent models of alcohol use. Humans with AUD have lower serum BDNF levels (Nubukpo et al. 2017; Zanardini et al. 2011), and lower BDNF in alcoholics is correlated with greater reported alcohol withdrawal severity (Heberlein et al. 2010). More recently, Garcia-Marchena et al. reported that low BDNF levels are still detected in abstinent alcoholics (Garcia-Marchena et al. 2017). In addition, in humans, rs6265 single point polymorphism (SNP) within the BDNF gene conferring a valine to methionine substitution in the prodomain of the BDNF protein, results in increased sensitivity to the anticipation of stress in healthy adults as well as to an increase in baseline levels of alcohol intake (Colzato et al. 2011). Furthermore, this polymorphism is also associated with an early onset of relapse in treatment-seeking alcoholics (Wojnar et al. 2009), and an increased likelihood to drink and respond to alcohol cues in adolescents (Nees et al. 2015). This polymorphism has been shown to alter the intracellular trafficking and packaging of proBDNF, affecting the activity-dependent secretion of the mature protein (Chen et al. 2004; Egan et al. 2003). In line with the human data, mice carrying the Val68/Met mutation consume alcohol despite negative consequences (Warnault et al. 2016). Furthermore, Val68/Met BDNF mice that consume alcohol despite the addition of quinine revert to moderate levels of drinking upon overexpression of WT BDNF in the mPFC or in response to the systemic administration of the BDNF activatior, LM22A-4 (Warnault et al. 2016). In sum, human and rodent data suggest that engagement of the STOP pathway via enhancement of BDNF may be beneficial in treating humans with AUD.

### GDNF

GDNF belongs to the transforming growth factor β (TGFβ) family of growth factors (Airaksinen and Saarma 2002; Ibanez and Andressoo 2017). GDNF was discovered in glioma cells (Lin et al. 1993) although the growth factor is mainly expressed in neurons (Pochon et al. 1997). GDNF acts through binding to the tyrosine kinase receptor, Ret, and the co-receptor, glycosyl-phosphatidylinositol-linked GDNF family receptor α1 (GFRα1) (Airaksinen and Saarma 2002; Ibanez and Andressoo 2017). Binding of GDNF to its receptors results in the activation of Ret which in turn induces the activation of the MAPK, PLCγ, and PI3K pathways (Airaksinen and Saarma 2002; Ibanez and Andressoo 2017). The majority of GDNF is produced in the striatum, whereas the majority of the Ret receptors are localized in the midbrain (Trupp et al. 1997), and GDNF is retrogradely transported by dopaminergic neurons from the striatum to the midbrain (Wang et al. 2010b; Tomac et al. 1995). GDNF plays an important role for the survival and maintenance of midbrain dopaminergic neurons (Lin et al. 1993; Pascual et al. 2008), as well as in the firing rate of dopaminergic neurons in the midbrain (Wang et al. 2010b; Yang et al. 2001). GDNF also plays a role in hippocampal synaptogenesis, differentiation of hippocampal and cortical GABAergic neurons, and the development of the olfactory system (Airaksinen and Saarma 2002; Ibanez and Andressoo 2017).

### GDNF and alcohol

Like early studies in the BDNF field, *Gdnf* HET mice were used to probe the role of the neurotrophic factor in the brain. Also like *Bdnf* HET mice (McGough et al. 2004), *Gdnf* HET mice consume more alcohol as compared to WT littermates. (Carnicella et al. 2009b). In addition, *Gdnf* HET mice display an enhanced alcohol- CPP as compared to their corresponding WT littermates (Carnicella et al. 2009b), implicating this endogenous growth factor in gating alcohol intake and reward.

Further work indicates that *Gdnf*, like *Bdnf* is also an alcohol responsive gene although the time-course of alcohol-dependent alterations of *GDNF* expression is different from *BDNF*. *GDNF* mRNA increases in the ventral tegmental area (VTA) following a brief (1 week) regime of 20%IA-2BC (Ahmadiantehrani et al. 2014a), which is not observed after a prolonged consumption (7 weeks) of 20% IA-2BC (Ahmadiantehrani et al. 2014a).

Next, a RNAi strategy was used to decipher the role of GDNF in alcohol-drinking behaviors. Interestingly, RNAi-mediated knockdown of *GDNF* in either the VTA or NAc of rats increases alcohol intake, enhances the onset of escalation of alcohol consumption, and magnifies relapse (Ahmadiantehrani et al. 2014a; Barak et al. 2015). Conversely, intra-VTA infusion of recombinant GDNF in the VTA reduces rats’ alcohol reward, consumption, and prevents relapse (Barak et al. 2011a; Barak et al. 2011b; Carnicella et al. 2009c; Carnicella et al. 2008). Further, viral-mediated overexpression of GDNF in the NAc or VTA prevents the development of excessive alcohol consumption in a 20% IA-2BC procedure (Barak et al. 2015). However, infusion of GDNF into the VTA fails to alter rat operant self-administration of sucrose (Carnicella et al. 2008), indicating that similar to BDNF, GDNF’s actions are specific for alcohol.

Also similar to BDNF, GDNF gates alcohol intake through the activation of ERK1/2, but not PI3K or PLC gamma (Carnicella et al. 2008). Unlike BDNF, GDNF-mediated reduction of alcohol intake is very rapid and occurs within minutes (Carnicella et al. 2008) suggesting that GDNF gates alcohol intake via a non-genomic mechanism. Furthermore, unlike BDNF, GDNF-mediated reduction of alcohol intake is long-lasting (Carnicella et al. 2008), which is mediated, at least in part, by the autoregulation of GDNF expression in the VTA (Barak et al. 2011a). Mechanistically, GDNF reduces excessive alcohol consumption by adjusting the firing rate of dopaminergic VTA neurons (Barak et al. 2015) and by normalizing withdrawal-induced reductions in NAc dopamine release (Barak et al. 2011b). Several lines of data suggest that AUD may be a result of a breakdown of the GDNF signaling pathway. For instance, *GDNF* levels are reduced in response to long-term alcohol intake in the VTA of rats (Ahmadiantehrani et al. 2014a), and a 50% reduction in *GDNF* levels in the VTA was associated with cue-induced alcohol seeking and drinking (Zipori et al. 2017). Finally, studies on the potential perturbation of the GDNF pathway in humans are limited. However, Heberlein et al. reported that human alcoholics exhibit reduced serum GDNF during both acute and protracted abstinence from alcohol, and that serum GDNF is negatively correlated with alcohol tolerance (Heberlein et al. 2010).

In sum, animal studies suggest that BDNF and GDNF are recruited in a number of brain regions to prevent the escalation of alcohol use, but, through various mechanisms, continued excessive alcohol consumption overwhelms the beneficial effects of the STOP pathways resulting in escalation of alcohol use as well as the development of other adverse phenotypes associated with AUD.

### Potential therapeutics targeting the BDNF and GDNF pathways

Below, we summarize potential therapeutics aimed at treating excessive alcohol consumption through the targeting of the BDNF and GDNF pathways including TrkB agonists and inhibitors of p75NTR as well as small molecules that serve as BDNF or GDNF mimetics (Fig. 2).

### Small molecules targeting the BDNF receptors

As described above, the BDNF receptors TrkB and p75NTR can be viewed as Yin and Yang in which TrkB serves as the transducer of a STOP mechanism (Jeanblanc et al. 2009; Jeanblanc et al., 2013) and p75NTR serves as a GO transducer (Darcq et al. 2016). Indeed, preclinical rodent studies suggest that targeting both receptors may be a useful strategy to combat phenotypes associated with AUD. Longo, Massa, and others have been developing small molecules targeting TrkB and p75NTR mainly for the treatment of neurological disorders (Longo and Massa 2013), and two of these compounds, LM22A-4 and LM11A31, have been used recently in preclinical AUD studies indicating their potential efficacy for the treatment of AUD. LM22A-4 was identified using computational modeling and virtual screening approach aimed at identifying a small molecule TrkB activator (Massa et al. 2010). Using *in vitro* assays in hippocampal neurons, LM22A-4 was shown to activate TrkB signaling (Massa et al. 2010), and the compound was shown to exhibit beneficial effects in animal models of Rett syndrome, Huntington’s disease (Schmid et al. 2012; Simmons et al. 2013), and stroke (Han et al. 2012). LM11A-31 was also identified in an *in silico* screen which was further characterized and found to bind to the p75NTR (Longo and Massa 2013). Animal studies have shown that LM11A31 may be developed for the treatment of Alzheimer’s disease (Knowles et al. 2013; Simmons et al. 2014; Yang et al. 2008), spinal cord injury (Tep et al. 2013), peripheral neuropathy (Friesland et al. 2014), and Huntington’s disease (Simmons et al. 2016). Using preclinical mouse models for AUD, we showed that a single systemic administration of LM11A31 or LM22A-4 significantly reduced mouse drinking of alcohol to a moderate level (Darcq et al. 2016; Warnault et al. 2016). Furthermore, the administration of LM22A-4 reduced compulsive alcohol intake in the BDNF Val68/Met mutant mice (Warnault et al. 2016). Importantly for drug development, the treatment of either compounds did not alter the consumption of water or sucrose (Darcq et al. 2016; Warnault et al. 2016). LM22A-4 has not been used in humans yet, and of note is a recent study which did not detect LM22A-4-dependent activation of TrkB receptor in cell culture models (Boltaev et al. 2017). However, clinical trials are currently underway to test the efficacy of LM11A31 in Alzheimer’s disease patients (https://clinicaltrials.gov/ct2/show/NCT03069014). Thus, these small molecules may be good candidates to proceed in clinical AUD studies.

### BDNF and GDNF mimetics

Another approach to target signaling within the STOP pathways is through small molecules that act on the level of gene transcription. Several examples of inducers of *BDNF* and *GDNF* transcription are outlined below.

*Memantine* is a non-competitive NMDA receptor inhibitor which was shown to reduce alcohol drinking and craving (Krishnan-Sarin et al. 2015; Krupitsky et al. 2007a), and to reduce withdrawal symptoms severity in alcoholics (Krupitsky et al. 2007b). Memantine was reported to reduce alcohol self-administration and craving in rats (Alaux-Cantin et al. 2015; Jeanblanc et al. 2014), and interestingly, Jeanblanc et al. reported that systemic administration of memantine increases BDNF expression in the dorsal striatum and mPFC of rats (Jeanblanc et al. 2014). Furthermore, Jeanblanc et al. showed that reduction of alcohol self-administration by memantine was attenuated by the inhibition of BDNF signaling (Jeanblanc et al. 2014). These data raise the possibility that other small molecules that target BDNF transcription and/or translation may be useful in treating AUD. Forthcoming results of a clinical trial led by Dr. Krishnan-Sarin on the interactive effects of naltrexone and memantine (https://clinicaltrials.gov/ct2/show/NCT01519063) will further develop our knowledge regarding the therapeutic value of the drug for the treatment of AUD.

*Ibogaine* is an alkaloid extracted from the root bark of a West African shrub, Tabernanthe Iboga. Ibogaine has hallucinogenic properties and as such is used in West Africa for religious rituals (Brown 2013). Ibogaine has been of interest in addiction research for decades because of its desirable actions to reduce withdrawal symptoms, drug intake of, as well as craving of multiple drugs of abuse in humans (Brown 2013). Strikingly, a single dose of ibogaine reduces drug craving for months (Noller et al. 2017; Sheppard 1994). An FDA phase 1 clinical trial was conducted in the 1990s. However, due to safety concerns, the FDA did not approve a large-scale phase 2 trial (Brown 2013). Ibogaine is, however, being used for the treatment of addiction of multiple drugs of abuse in clinics outside of the USA (Brown 2013). Recent studies conducted in Mexico and New Zealand raised again the possibility that ibogaine may be a possible treatment for opiate addition. Specifically, Brown et al. and Nollan et al. reported that ibogaine was effective in blocking acute and long-term opiate withdrawal symptoms (Noller et al. 2017; Sheppard 1994).

Although the beneficial actions of ibogaine are numerous, serious safety concerns and the fact that the drug is hallucinogenic significantly reduce enthusiasm for the development of ibogaine *per se* for the treatment of addiction. In an attempt to separate the desirable actions of ibogaine from the undesirable side effects, we conducted a microarray study in rats to identify potential downstream targets through which ibogaine exerts its beneficial actions on alcohol intake. We found that ibogaine as well as its derivative noribogaine increase *GDNF* expression in a dopaminergic-like cell line and in the VTA of rats (Carnicella et al. 2010; He et al. 2005). We further showed that ibogaine reduces alcohol self-administration and relapse through GDNF signaling in the VTA (He et al. 2005). Specifically, we found that blocking GDNF signaling in the VTA inhibited ibogaine-dependent reduction of alcohol self-administration (He et al. 2005). We further showed that ibogaine produces a long-lasting attenuation of alcohol self-administration (He et al. 2005), by inducing a positive autoregulatory loop by which *GDNF* transcription is controlled by the growth factor itself (He and Ron 2006). Finally, ibogaine prevented the alcohol-dependent increase in tyrosine hydroxylase through a mechanism that required GDNF in a dopaminergic-like cell line (He and Ron 2008). Strucure-function studies are required to identify ibogaine derivatives that will not posses the undesirable actions such as hallucination and neurotoxicity but will retain the desirable GDNF-mimetic property of the drug.

Cabergoline is another small molecule which increases *GDNF* expression and activates GDNF signaling in cultured cells and in vivo (Carnicella et al. 2009a). Cabergoline (Dostinex, Cabaser) is an FDA-approved drug being used for the treatment of hyperprolactinemia (Bogazzi et al. 2008; Webster et al. 1994), and interestingly, a pilot study conducted in humans suggests that cabergoline reduces cocaine use (Shoptaw et al. 2005). We reported that cabergoline acts similarly to ibogaine. Incubation of cabergoline in cultured cells and systemic administration of this drug in rats increased the expression of *GDNF*, resulting in the activation of the GDNF receptor, Ret (Carnicella et al. 2009a). We further showed that cabergoline reduces alcohol drinking and seeking and importantly, the drug reduced rat alcohol self-administration in a model of relapse without affecting sucrose intake (Carnicella et al. 2009a). Finally, we showed that cabergoline-dependent increase in *GDNF* expression and reduction of alcohol intake were abolished in *Gdnf* HET mice (Carnicella et al. 2009a) indicating that cabergoline reduces alcohol-drinking behaviors via GDNF. Cabergoline acts as a dopamine 2 receptor agonist (Newman-Tancredi et al. 2002); however, as outlined above, the effect of cabergoline on alcohol-related behaviors is likely to be dependent solely on the GDNF system (Carnicella et al. 2009a).

Together, these examples show that AUD may be treated by drugs that act through the activation of STOP pathway signaling pathways, and/or through the induction of gene expression of players within the STOP pathway.

## GO pathways facilitate excessive alcohol consumption

Opposing the STOP pathways are the GO pathways, a set of molecular machineries which react to alcohol exposure by initiating changes that promote the escalation of drinking from moderate to excessive levels (Fig. 1). Like the STOP pathways, components of the GO pathways are distributed throughout corticostriatal and amygdalar brain regions. Below, we give two examples of GO signaling which centers around two kinases: Fyn and the mammalian target of rapamycin (mTOR). We then discuss the potential use of inhibitors of Fyn and mTOR signaling for the treatment of AUD.

### Fyn

Much of alcohol’s influence on behavior centers on the glutamatergic system (Goodwani et al. 2017; Hwa et al. 2017). Of note is the ionotropic *N*-methyl-d-aspartate receptor (NMDAR), a critical component on neuroplasticity and long-term memory formation (Malenka and Nicoll 1993), which plays an important role in various phenotypes associated with AUD (Morisot and Ron 2017; Ron and Wang 2009). The major regulatory NMDAR subunits are GluN2A and GluN2B (Ogden and Traynelis 2011), which contain long cytoplasmic tails that are subjected to posttranslational modifications such as phosphorylation (Ron 2004; Trepanier et al. 2012). One of the major kinases phosphorylating the GluN2B subunit is the tyrosine kinase, Fyn (Trepanier et al. 2012). The phosphorylation of GluN2B by Fyn enhances channel function (Trepanier et al. 2012; Yaka et al. 2003, 2002) through the membranal retention of GluN2B (Dunah et al. 2004; Nakazawa et al. 2001; Prybylowski et al. 2005). Fyn contributes to excitatory and inhibitory synaptic transmission (Chattopadhyaya et al. 2013; Hildebrand et al. 2016; Ohnishi et al. 2011) and to learning and memory (Grant et al. 1992; Kojima et al. 1997). Fyn, a member of the Src family of tyrosine kinases, exists in either an active or an inactive conformation (Engen et al. 2008). Fyn activity is regulated by two phosphatases: protein tyrosine phosphatase alpha (PTPα) and the striatal-enriched tyrosine phosphatase (STEP). Activation of Fyn is achieved through the dephosphorylation of tyrosine residue Tyr527 by PTPα (Bhandari et al. 1998; Ponniah et al. 1999; Vacaresse et al. 2008). Full activation of the kinase requires the autophosphorylation at Tyrosine 417 (Engen et al. 2008) and Fyn is inactivated via the dephosphorylation of Tyr417 by STEP (Goebel-Goody et al. 2012).

### mTOR

mTOR is a serine and threonine kinase which is compartmentalized in two distinct multi-protein complexes, defined as mTOR complex 1 (mTORC1) and mTOR in complex 2 (mTORC2) (Ma and Blenis 2009; Saxton and Sabatini 2017; Zoncu et al. 2011). mTORC1 and mTORC2 complexes exclusively include the adaptor proteins regulatory-associated protein of mTOR (RAPTOR) found in mTORC1 and rapamycin-insensitive companion of mTOR (RICTOR) found in mTORC2 (Ma and Blenis 2009; Saxton and Sabatini 2017; Zoncu et al. 2011). RAPTOR and RICTOR are required for the activity of mTORC1 and mTORC2, respectively (Ma and Blenis 2009; Saxton and Sabatini 2017; Zoncu et al. 2011). Although mTOR bears the same kinase activity in both complexes, the unique multiprotein network comprising the kinase results in disparate mTORC1 and mTORC2 substrates and downstream cellular functions (Saxton and Sabatini 2017). Another important distinction between mTORC1 and mTORC2 is the sensitivity to rapamycin. Rapamycin is a selective inhibitor of mTORC1 that does not affect the activity of mTORC2 (Li et al. 2014).

mTORC1 is activated through the activation of ERK1/2 or its downstream kinase, the 90 kDa ribosomal S6 kinase (RSK), as well as AKT (also known as PKB) (Ma and Blenis 2009; Mendoza et al. 2011; Zoncu et al. 2011). Much less is known about the mechanisms enabling mTORC2 activation. mTORC2 is activated through the activation of growth factor signaling and through its association with the ribosomes (Oh and Jacinto 2011; Zinzalla et al. 2011).

mTORC1 phosphorylates the S6 ribosomal kinase 1 (S6K1) and the eukaryotic translation initiation factor-4E binding protein (4E-BP), both of which are part of the ribosomal machinery (Costa-Mattioli et al. 2009; Ma and Blenis 2009). Phosphorylation of S6K1 and 4E-BP controls the initiation and elongation of translation of a subset of mRNAs displaying a 5′ terminal oligopyrimidine (TOP) motif (Thoreen et al. 2012; Zoncu et al. 2011). Specifically, mTORC1-induced phosphorylation of 4E-BP allows the interaction of the eukaryotic translation initiation factor 4E (eIF4E) with eIF4G at the 5′ cap structure of mRNAs, a process that is critical for translation initiation (Ma and Blenis 2009). mTORC1 activation also increases translational rate by increasing the production of ribosomal proteins and translation factors (Liao et al. 2007), as well as by inducing the transcription of ribosomal RNAs (rRNAs) (Zoncu et al. 2011). mTORC2’s well-characterized substrates are AKT, serum, and glucocorticoid-induced protein kinase 1 (SGK1) and protein kinase C α (PKC α) (Oh and Jacinto 2011; Zinzalla et al. 2011). In the adult brain, mTORC1 is a critical mediator of synaptic plasticity (Lipton and Sahin 2014) including the induction of the late phase of long-term potentiation (Cammalleri et al. 2003; Stoica et al. 2011; Tang et al. 2002), due to its capacity to promote local dendritic protein synthesis (Buffington et al. 2014; Gobert et al. 2008; Hoeffer and Klann 2010; Stoica et al. 2011). Behaviorally, mTORC1 contributes to memory processes. For example, local inhibition of mTORC1 in the rat mPFC by rapamycin results in long-term memory deficits of trace fear memory examined days after conditioning training, without impacting short-term trace fear memory and object recognition memory (Sui et al. 2008). Rapamycin was shown to also impair novel object recognition when infused into the basolateral amygdala (BLA) or dorsal hippocampus of rats (Jobim et al. 2012; Myskiw et al. 2008). Moreover, mTORC1 activity is also implicated in the consolidation and reconsolidation of fear- and drug-related memories (Blundell et al. 2008; Gafford et al. 2011; Glover et al. 2010; Lin et al. 2014; Mac Callum et al. 2014; Slipczuk et al. 2009; Stoica et al. 2011). Conversely, mice with constitutively active mTORC1 via FK506 binding protein 12 (FKBP12) (a cis/trans peptidyl prolyl isomerase (Tong and Jiang 2015)) knockout, display increased long-term contextual fear memory retention and compulsive-like perseverative behaviors in tasks such as the marble burying assay, object recognition task, Morris water maze, and Y maze reversal task (Hoeffer et al. 2008). However, cued memory and short-term contextual memories were not impacted, again indicating that mTORC1 activity may be selective for long-term memories and compulsive behaviors (Hoeffer et al. 2008). Much less is known about the role of mTORC2 in the CNS. mTORC2 contributes to F-actin assembly in the hippocampus (Huang et al. 2013), and in the DMS (Laguesse et al. 2018). mTORC2 in the hippocampus was also shown to contribute to late phase long-term potentiation (L-LTP) (Huang et al. 2013), and to memory consolidation (Huang et al. 2013). mTORC2 was also reported to contribute to the membrane expression of the dopamine D2 receptors (D2R) in the striatum (Dadalko et al. 2015), and to control the size and shape of cerebellar Purkinje cells (Thomanetz et al. 2013). Due to its influence on neuroplasticity, malfunction of mTORC1 and to some extent also mTORC2 have been implicated in numerous psychiatric disorders (Costa-Mattioli and Monteggia 2013; Lipton and Sahin 2014) including addiction (Costa-Mattioli and Monteggia 2013; Dayas et al. 2012; Jernigan et al. 2011; Neasta et al. 2014).

### Fyn, mTOR, and alcohol

Unlike BDNF and GDNF which are examples of genes participating in the “STOP” pathways that gate the level of alcohol intake (Fig. 1), the kinases Fyn and mTOR are examples of genes that participate in neuroadaptations that underlie the development and maintenance of maladaptive alcohol-drinking and seeking behaviors and hence are part of the GO pathways (Fig. 1).

### Alcohol activates Fyn and mTOR

Repeated cycles of binge drinking and withdrawal result in Fyn activation specifically in the DMS (Darcq et al. 2014; Gibb et al. 2011; Wang et al. 2010a), whereas mTORC1 is specifically activated in the NAc (Beckley et al. 2016; Laguesse et al. 2016; Neasta et al. 2010) and in the orbitofrontal cortex (OFC) (Laguesse et al. 2016) of rodents. Similar to Fyn, chronic alcohol intake activates mTORC2 specifically in the DMS (Laguesse et al. 2018). The molecular mechanisms of Fyn and mTORC1 activation in the DMS and NAc, respectively, are well-characterized. Specifically, Fyn is activated by alcohol in the DMS through the recruitment of PTPα (Ben Hamida et al. 2013), and via the inhibition of STEP (Darcq et al. 2014). mTORC1 is activated by alcohol through the activation on the small G protein H-Ras (Ben Hamida et al. 2012), which then leads to the activation of the PI3K/AKT pathway (Neasta et al. 2011). Both Fyn and mTORC1 signaling in the DMS and NAc, respectively, are activated through the stimulation of dopamine D1 receptor (D1R) (Beckley et al. 2016; Phamluong et al. 2017) raising the possibility that both pathways are activated in their respective brain region through alcohol-dependent dopamine release and the activation of D1R. Support of this possibility stems from the finding that mTORC1 is activated by alcohol specifically in D1R-expressing neurons in the NAc (Beckley et al. 2016). Interestingly, Fyn and mTORC1 activation by alcohol further depends on the amount of alcohol consumed, and the type of alcohol exposure. Specifically, intermittent access to 20% alcohol activates both kinases, whereas continuous access to 10% alcohol does not (Laguesse et al. 2016; Morisot and Ron 2017). Finally, reconsolidation of the memory of alcohol seeking (e.g., the smell or taste of alcohol) produces a robust activation of mTORC1 in the CeA as well as in the mPFC and OFC but not in the NAc (Barak et al. 2013). How alcohol activates mTORC2 in the DMS, or how a brief reconsolidation period activates mTORC1 in selected brain regions has yet to be determined.

### Cellular and behavioral consequences of Fyn and mTOR activation by alcohol

As stated above, the major substrate of Fyn in the brain is GluN2B (Trepanier et al. 2012); indeed, GluN2B phosphorylation is detected in response to intermittent access to 20% alcohol in the same brain region where Fyn is activated, namely the DMS (Ben Hamida et al. 2013; Darcq et al. 2014; Wang et al. 2010a). The phosphorylation of GluN2B by alcohol at the synaptosomal membrane is long-lasting and is detected even after 24 hours of withdrawal (Ben Hamida et al. 2013; Darcq et al. 2014; Wang et al. 2010a). *Ex vivo* exposure of DMS neurons to alcohol produces a long-lasting facilitation of NMDAR activity which is both Fyn and GluN2B-dependent (Wang et al. 2007; Wang et al. 2010a). Interestingly, the activity of NMDAR in the DMS of rats is robustly elevated in response to chronic voluntary consumption of alcohol and is still detected even 9 days after withdrawal (Wang et al. 2010a). As stated above, PTPα is necessary for alcohol-dependent Fyn activation (Gibb et al. 2011), and in support to this conclusion, PTPα knockdown of the DMS prevented alcohol-dependent GluN2B phosphorylation (Ben Hamida et al. 2013). The opposite holds true as well; STEP inhibition by alcohol is also required for Fyn activation (Darcq et al. 2014), and downregulation of STEP in the DMS enhances alcohol-induced Fyn activation and GluN2B phosphorylation (Darcq et al. 2014). GluN2B phosphorylation increases NMDAR function thereby enabling calcium entry into the cell (Trepanier et al. 2012). Calcium is a second messenger necessary for the activation of calcium/calmodulin-dependent protein kinase type II (CaMKII) (Coultrap and Bayer 2012). While the effect of alcohol consumption on CaMKII activity in the DMS has not been evaluated, alcohol exposure increases CaMKII activity in the CeA and mPFC of rodents (Natividad et al. 2017; Salling et al. 2016). CaMKII phosphorylation of α-amino-3-hydroxy-5-methyl-4-isoxazolepropionic acid receptor (AMPAR) subunits, GluA1 and GluA2, is required for the forward trafficking of the subunits to the plasma membrane (Malinow and Malenka 2002), and as expected, alcohol drinking leads to increased membrane localization of AMPAR in the DMS (Wang et al. 2012). Together, these data suggest that the activation of Fyn by alcohol produces long-lasting molecular and cellular adaptations in the DMS. Pharmacological and molecular manipulations of genes within the Fyn pathway produced significant alterations of alcohol-drinking behaviors (Morisot and Ron 2017). For instance, knockdown of PTPα in the DMS reduced the consumption of higher concentrations of alcohol: 6, 10, and 20%, but not 3% (Ben Hamida et al. 2013). This indicates that preventing the alcohol-induced activation of Fyn alters the development of excessive alcohol consumption. Conversely, knockdown of STEP in the DMS increased alcohol intake of mice (Darcq et al. 2014), and *STEP* knockout (KO) mice consume more alcohol than their wild-type counterparts (Legastelois et al. 2015). Interestingly, STEP KO mice also display reduced conditioned place aversion (CPA) to lithium chloride and are less sensitive to quinine adulteration, which robustly reduces alcohol consumption in control animals (Legastelois et al. 2015). This indicates that STEP influences alcohol consumption by making the animal less sensitive to aversive sensations. Pharmacological inhibition of Fyn or GluN2B with PP2 or ifenprodil, respectively, in the DMS, but not NAc or DLS, reduced operant responding for alcohol while not impacting overall locomotor behavior or motivation for natural rewards such as sucrose (Wang et al. 2007; Wang et al. 2010a). Importantly for the therapeutic potential of drugs aimed at Fyn signaling, intra-DMS inhibition of Fyn also reduced the reinstatement of alcohol seeking and thus relapse (Wang et al. 2010a) using a previously developed method of reinstating alcohol seeking via exposure to alcohol odor and taste (Carnicella et al. 2014). Additionally, mice expressing an inactive form of CaMKII exhibit a delay in moderate alcohol consumption, indicating that activity of CaMKII contributes to alcohol reward (Easton et al. 2013). Additionally, alcohol-induced phosphorylation of CaMKII in the dorsal mPFC was associated with an increase in perseverative behavior in a rodent operant self-administration paradigm (Natividad et al. 2017). Finally, intra-DMS administration of the AMPAR antagonist NBQX decreased operant self-administration of alcohol (Wang et al. 2012). Together, these data indicate that activation of the Fyn/GluN2B pathway by alcohol leads to the induction of synaptic plasticity which in turn drives and maintains alcohol-related behaviors.

As stated above, mTORC1 activation initiates the dendritic translation of proteins (Buffington et al. 2014; Gobert et al. 2008; Hoeffer and Klann 2010; Stoica et al. 2011), and as such, the kinase plays a critical role in synaptic plasticity learning and memory (Lipton and Sahin 2014). Plasticity is critical for the development of drug-related behaviors (Hyman et al. 2006), and we generated data to suggest that mTORC1 in the NAc, and mTORC2 in the DMS play a critical role in alcohol-dependent synaptic and structural plasticity. Specifically, we found that heavy alcohol consumption in rodents leads to the translation of synaptic proteins in the NAc such as the GluA1 subunit of the AMPAR, the scaffolding proteins, HOMER, postsynaptic density 95 (PSD-95), and ProSAP2-interacting protein 1 (Prosapip1), as well as Arc and collapsin response mediator protein 2 (CRMP 2), (Beckley et al. 2016; Laguesse et al. 2017; Liu et al. 2017; Neasta et al. 2010). Interestingly, a brief reconsolidation period also led to an increase in the protein levels of GluA1, Arc and PSD95 in the amygdala, OFC, and mPFC of rats (Barak et al. 2013). The cellular consequences of mTORC1 activation and the dendritic translation are the strengthening of excitatory neurotransmission in D1R neurons in the NAc in mice in response to the first alcohol drink (Beckley et al. 2016), and to the increase in the activity of GluA2-lacking AMPAR in response to 7 weeks of heavy drinking (Laguesse et al. 2017). Further data indicate that upregulation in the level of CRMP2 and Prosapip1 by alcohol results in profound morphological alterations. Specifically, CRMP2 is a tubulin binding protein (Quach et al. 2015), and we recently showed that excessive alcohol intake increases the binding of CRMP2 to tubulin which in turn causes microtubule assembly of NAc neurons (Liu et al. 2017). Furthermore, we found that excessive alcohol intake increases the amount of filamentous (F) actin and decreases the amount of globular (G) actin through a mechanism that requires Prosapip1 (Laguesse et al. 2017), a component of the scaffolding machinery in the postsynaptic densities of excitatory spines (Wendholt et al. 2006). We further showed that the consequence of the F-actin polymerization by alcohol is an increased number of mushroom spines in the NAc and a decrease in the number of immature thin spines (Laguesse et al. 2017). Interestingly, mTORC2 in the DMS is also part of the molecular machinery that produces F-actin assembly and maturation of dendritic spines in response to heavy alcohol intake in mice (Laguesse et al. 2018).

The activation of mTORC1 and the consequent translation of CRMP2 and Prosapip1 produce profound alterations in animal drinking behaviors. Specifically, systemic administration of the selective mTORC1 inhibitors rapamycin or rapalink-1 attenuates rat operant self-administration of alcohol in rats (Neasta et al. 2010) and voluntary alcohol intake in a 2-BC paradigms in rodents (Beckley et al. 2016; Morisot et al. 2017; Neasta et al. 2010) without altering sucrose (Neasta et al. 2010) or saccharine intake (Morisot et al. 2017). Furthermore, a single systemic administration of rapalink-1 produced very long-lasting inhibitory effects on alcohol intake which lasted for at least 14 days post-drug administration (Morisot et al. 2017). Importantly, confirming the role of mTORC1 in the NAc in alcohol-drinking behaviors, intra-NAc administration of rapamycin reduces binge consumption of alcohol (Neasta et al. 2010). Furthermore, rapamycin administration also inhibited mice alcohol place preference and sensitization (Neasta et al. 2010). Finally, systemic administration of lacosamide, a drug that inhibits CRMP2 function in part by directly binding to tubulin and disrupting microtubules assembly (Beyreuther et al. 2007; Wilson and Khanna 2015), or viral-mediated knockdown of CRMP2 and Prosapip1 in the NAc, significantly reduced alcohol intake in mice (Laguesse et al. 2017; Liu et al. 2017), without altering saccharine intake (Laguesse et al. 2017; Liu et al. 2017). Further tests indicated that similar to rapamycin, attenuation of Prosapip1 levels in the NAc inhibited alcohol CPP (Laguesse et al. 2017). Finally, systemic or intra-CeA pretreatment with rapamycin impairs reconsolidation during an alcohol reactivation test, as evidenced by a reduction in alcohol seeking during on the next day and subsequently during a reacquisition test (Barak et al. 2013). The influence of this pharmacological manipulation was observed even 14 days later (Barak et al. 2013), displaying the effect that mTORC1 signaling plays in relapse and long-term memories alcohol seeking. Together, these data suggest that mTORC1 signaling plays a crucial role in alcohol reward, in the development and maintenance of alcohol intake, and in mechanisms underlying relapse. Very recent data suggest that mTORC2 in the DMS also plays a role in alcohol intake as the knockdown of RICTOR, an essential component in the mTORC2 complex (Ma and Blenis 2009; Saxton and Sabatini 2017; Zoncu et al. 2011), in the DMS reduced, whereas the systemic administration of the mTORC2 activator A-443654 increased alcohol intake in mice (Laguesse et al. 2018). Together, these findings indicate that the therapeutic benefit of mTORC1 inhibition on AUD phenotypes may be the result of destabilizing synaptic plasticity related to alcohol memories.

### The GO pathway in humans

Human studies provide a potential link between genetic mutations within the GO pathways and AUD, although it is unknown whether any of these mutations confirm a gain or loss of function. Specifically, single nucleotide polymorphisms (SNPs) within the Fyn gene have been linked to increased risk of AUD development and increased severity of the disorder once it has developed (Ishiguro et al. 2000; Pastor et al. 2009; Schumann et al. 2003). The Fyn gene was also found in a gene network associated with alcohol dependence (Han et al. 2013). Finally, mutations within mTOR, other enzymes within the translational machinery, and HOMER and AMPAR subunits have been linked to increased alcohol use in humans (Meyers et al. 2015).

## Medication development

### Potential medications targeting the Fyn pathway

Fyn is a member of the Src family of tyrosine kinases (Engen et al. 2008), and the Src and Fyn inhibitor AZD0530 (saracatinib) represents a potential new therapeutic to treat AUD. Phase I and II clinical trials in cancer and Alzheimer's disease (AD) research showed that oral administration of AZD0530 is safe, well-tolerated in patients, and with considerable high brain penetration (Baselga et al. 2010; Molina et al. 2014; Nygaard et al. 2015). Therefore, testing the efficacy of the drug in humans suffering from AUD is of great interest.

## Potential medications targeting mTOR

### Rapamycin and Rapalink-1

As detailed above, preclinical animal studies indicate that the mTORC1 inhibitors, rapamycin and its analog rapalink-1, exhibit very desirable actions for the treatment of alcohol-related phenotypes. Rapamycin (sirolimus) is a macrolide antibiotic isolated from a bacteria found in the soil of the Eastern Island, Rapu Anu. Rapamycin is as an allosteric-specific inhibitor of mTORC1, which, in complex with FKBP12 protein, binds to the FRB (FKBP12-rapamycin binding) domain of the kinase (Dowling et al. 2010; Yip et al. 2010). This interaction is thought to modify mTORC1 conformation, which in turn not only weakens the integrity of the kinase complex but also prevents the association of its catalytic site with its substrates (Yip et al. 2010). Rapalink-1 is a rapamycin analog in which rapamycin is covalently linked with a mTOR kinase inhibitor (Rodrik-Outmezguine et al. 2016). In addition to being a selective mTORC1 inhibitor, rapamycin is a potent immunosuppressant and is an FDA-approved drug used post-organ transplantation surgery to prevent organ rejection (Li et al. 2014). Rapamycin has also been clinically tested for indications such as cancer and neurodegenerative diseases (Bove et al. 2011; Chiarini et al. 2015; Ilagan and Manning 2016). And, lending to the potential for mTORC1 as a therapeutic target for AUD, the actions of rapamycin and rapalink-1 were specific for alcohol and did not generalize to other rewarding substances, sucrose, and saccharine in preclinical animal models (Morisot et al. 2017; Neasta et al. 2010). Furthermore, rapamycin administration did not alter quinine intake in rodents, nor did it alter rodents' locomotor coordination, place preference or aversion (Barak et al. 2013; Neasta et al. 2010). Also of great promise is the finding that a single administration of rapalink-1 produces very long lasting actions - it reduces alcohol intake and relapse for at least 2 weeks (Barak et al. 2013; Morisot et al. 2017). Therefore, one can speculate that only a few doses of the drug will be required to reduce or eliminate craving during a sensitive period right after rehabilitation and this would sidestep issues of immunosuppression. Having said that, the development of new generations of mTOR inhibitors which inhibit both mTORC1 and mTORC2 without immunosuppressant side effects may be ideal new therapeutic approach for the treatment of AUD.

### Lacosamide - a CRMP 2 inhibitor

Lacosamide is an FDA-approved drug for the treatment of epilepsy (Krauss et al. 2012). Lacosamide directly interacts with CRMP2 and inhibits CRMP2 binding to tubulin (Beyreuther et al. 2007; Wilson and Khanna 2015). As stated above, CRMP2 is one of mTORC1’s downstream targets (Liu et al. 2017). Systemic administration of lacosamide significantly reduces binge drinking of alcohol in both mice and rats without altering water or sucrose intake, or changing locomotion and basal anxiety-like behavior (Liu et al. 2017). Furthermore, more recent studies indicate that the lacosamide decreases the reinstatement of alcohol place preference (Ben Hamida et al. In revision), suggesting that the drug can also be used to prevent relapse. Together, these findings in preclinical models suggest that lacosamide may be useful for the treatment of AUD.

## Conclusions and implications for the treatment of alcohol use disorder in humans

We described herein examples of intracellular signaling pathways within the corticostriatal and amygdalar circuitries that contribute to mechanisms that underlie both the gating (STOP pathways) and the initiation and/or maintenance (GO pathways) of excessive alcohol drinking (Fig. 1). It is of interest to note that most of the signaling cascades described herein are activated by alcohol in separate brain regions and that the mechanism of activation and the downstream cellular events that drive the behavioral outcomes are quite different. Furthermore, although the majority of signaling cascades described herein are altered in response to either moderate or excessive alcohol intake but not both, an exception is CaMKII. CamKII is activated in the CeA in response to moderate intake of alcohol (Salling et al. 2016), whereas NMDAR the transducer of CAMKII signaling, is activated in the DMS in response to excessive alcohol intake (Wang et al. 2010a). Furthermore, the potential crosstalk between the STOP and GO intracellular pathways is unknown and is an important direction for future research. Also of interest is the observation that although these molecular signaling pathways are unique, each of them is sufficient but not necessary for the gating or development of AUD. This conclusion stems from the fact that the activation of different components of the STOP pathways or inhibition of different signaling molecules within the GO pathways results in the exact same outcome, e.g., the reversal of alcohol drinking from an excessive uncontrolled state to moderate levels of intake. It is also important to note that none of these signaling cascades influence the natural reward circuitry, as manipulating them does not alter the intake of other rewarding substances. This information is of great importance for the translational implications of these findings, as inhibiting them does not seem to produce, at least in animal studies, undesirable side effects such as anhedonia. The signaling cascades described herein are also examples of how basic molecular neuroscience research can turn into fruitful translational research that results in numerous exciting new drugs and/or drug targets for the treatment of AUD. The potential new drugs that were identified by the basic science research can be divided into two classes of compounds: those that are already FDA-approved such as rapamycin and lacosamide and those that are in various phases of clinical trials such as LM11A31 and saracatinib (Fig. 2). However, in order to test the utility of these candidates for the treatment of AUD, human preclinical studies are of great need. Finally, based on the research that has been conducted up till now (Neasta et al. 2014), mTORC1 inhibitors may be useful for the treatment of not only AUD but other drugs of abuse as well.

## References

[CR1] Ahmadiantehrani S, Barak S, Ron D (2014). GDNF is a novel ethanol-responsive gene in the VTA: implications for the development and persistence of excessive drinking. Addict Biol.

[CR2] Ahmadiantehrani S, Warnault V, Legastelois R, Ron D (2014b) From signaling pathways to behavior: the light and dark sides of alcohol. In: Nohrona ABC, Cui C, Harris RA, Crabbe JC (eds) Neurobiology of alcohol dependence. Elsevier, pp 155–171

[CR3] Airaksinen MS, Saarma M (2002). The GDNF family: signalling, biological functions and therapeutic value. Nat Rev Neurosci.

[CR4] Alaux-Cantin S, Buttolo R, Houchi H, Jeanblanc J, Naassila M (2015). Memantine reduces alcohol drinking but not relapse in alcohol-dependent rats. Addict Biol.

[CR5] Andero R, Choi DC, Ressler KJ (2014). BDNF-TrkB receptor regulation of distributed adult neural plasticity, memory formation, and psychiatric disorders. Prog Mol Biol Transl Sci.

[CR6] Autry AE, Monteggia LM (2012). Brain-derived neurotrophic factor and neuropsychiatric disorders. Pharmacol Rev.

[CR7] Bahi A, Dreyer JL (2013). Striatal modulation of BDNF expression using microRNA124a-expressing lentiviral vectors impairs ethanol-induced conditioned-place preference and voluntary alcohol consumption. Eur J Neurosci.

[CR8] Barak S, Ahmadiantehrani S, Kharazia V, Ron D (2011a) Positive autoregulation of GDNF levels in the ventral tegmental area mediates long-lasting inhibition of excessive alcohol consumption. Transl Psychiatry 1 10.1038/tp.2011.57 PMC3253655 22238721

[CR9] Barak S, Carnicella S, Yowell QV, Ron D (2011). Glial cell line-derived neurotrophic factor reverses alcohol-induced allostasis of the mesolimbic dopaminergic system: implications for alcohol reward and seeking. J Neurosci.

[CR10] Barak S, Liu F, Ben Hamida S, Yowell QV, Neasta J, Kharazia V, Janak PH, Ron D (2013). Disruption of alcohol-related memories by mTORC1 inhibition prevents relapse. Nat Neurosci.

[CR11] Barak S, Wang J, Ahmadiantehrani S, Ben Hamida S, Kells AP, Forsayeth J, Bankiewicz KS, Ron D (2015). Glial cell line-derived neurotrophic factor (GDNF) is an endogenous protector in the mesolimbic system against excessive alcohol consumption and relapse. Addict Biol.

[CR12] Bartel DP (2004). MicroRNAs: genomics, biogenesis, mechanism, and function. Cell.

[CR13] Baselga J, Cervantes A, Martinelli E, Chirivella I, Hoekman K, Hurwitz HI, Jodrell DI, Hamberg P, Casado E, Elvin P (2010). Phase I safety, pharmacokinetics, and inhibition of SRC activity study of saracatinib in patients with solid tumors. Clin Cancer Res.

[CR14] Bazov I, Sarkisyan D, Kononenko O, Watanabe H, Yakovleva T, Hansson AC, Sommer WH, Spanagel R, Bakalkin G (2018) Dynorphin and kappa-opioid receptor dysregulation in the dopaminergic reward system of human alcoholics. Mol Neurobiol 10.1007/s12035-017-0844-4 PMC6061161 29383684

[CR15] Bazov et al. 10.1007/s12035-017-0844-4. [Epub ahead of print]

[CR16] Beckley JT, Laguesse S, Phamluong K, Morisot N, Wegner SA, Ron D (2016). The first alcohol drink triggers mTORC1-dependent synaptic plasticity in nucleus accumbens dopamine D1 receptor neurons. J Neurosci.

[CR17] Bekinschtein P, Cammarota M, Medina JH (2014). BDNF and memory processing. Neuropharmacology.

[CR18] Ben Hamida S, Darcq E, Wang J, Wu S, Phamluong K, Kharazia V, Ron D (2013). Protein tyrosine phosphatase alpha in the dorsomedial striatum promotes excessive ethanol-drinking behaviors. J Neurosci.

[CR19] Ben Hamida S, Neasta J, Lasek AW, Kharazia V, Zou M, Carnicella S, Janak PH, Ron D (2012). The small G protein H-Ras in the mesolimbic system is a molecular gateway to alcohol-seeking and excessive drinking behaviors. J Neurosci.

[CR20] Beyreuther BK, Freitag J, Heers C, Krebsfanger N, Scharfenecker U, Stohr T (2007). Lacosamide: a review of preclinical properties. CNS Drug Rev.

[CR21] Bhandari V, Lim KL, Pallen CJ (1998). Physical and functional interactions between receptor-like protein-tyrosine phosphatase alpha and p59fyn. J Biol Chem.

[CR22] Bjorkholm C, Monteggia LM (2016). BDNF—a key transducer of antidepressant effects. Neuropharmacology.

[CR23] Blundell J, Kouser M, Powell CM (2008). Systemic inhibition of mammalian target of rapamycin inhibits fear memory reconsolidation. Neurobiol Learn Mem.

[CR24] Bogazzi F, Buralli S, Manetti L, Raffaelli V, Cigni T, Lombardi M, Boresi F, Taddei S, Salvetti A, Martino E (2008). Treatment with low doses of cabergoline is not associated with increased prevalence of cardiac valve regurgitation in patients with hyperprolactinaemia. Int J Clin Pract.

[CR25] Boltaev U, Meyer Y, Tolibzoda F, Jacques T, Gassaway M, Xu Q, Wagner F, Zhang YL, Palmer M, Holson E, Sames D (2017). Multiplex quantitative assays indicate a need for reevaluating reported small-molecule TrkB agonists. Sci Signal.

[CR26] Bove J, Martinez-Vicente M, Vila M (2011). Fighting neurodegeneration with rapamycin: mechanistic insights. Nat Rev Neurosci.

[CR27] Boyce JM, Risinger FO (2000). Enhancement of ethanol reward by dopamine D3 receptor blockade. Brain Res.

[CR28] Boyce JM, Risinger FO (2002). Dopamine D3 receptor antagonist effects on the motivational effects of ethanol. Alcohol.

[CR29] Brown TK (2013). Ibogaine in the treatment of substance dependence. Curr Drug Abuse Rev.

[CR30] Brown TK, Alper K (2017) Treatment of opioid use disorder with ibogaine: detoxification and drug use outcomes. Am J Drug Alcohol Abuse:1–13 10.1080/00952990.2017.1320802 28541119

[CR31] Buckley PF, Mahadik S, Pillai A, Terry A (2007). Neurotrophins and schizophrenia. Schizophr Res.

[CR32] Buffington SA, Huang W, Costa-Mattioli M (2014). Translational control in synaptic plasticity and cognitive dysfunction. Annu Rev Neurosci.

[CR33] Cammalleri M, Lutjens R, Berton F, King AR, Simpson C, Francesconi W, Sanna PP (2003). Time-restricted role for dendritic activation of the mTOR-p70S6K pathway in the induction of late-phase long-term potentiation in the CA1. Proc Natl Acad Sci U S A.

[CR34] Carnicella S, Ahmadiantehrani S, He DY, Nielsen CK, Bartlett SE, Janak PH, Ron D (2009). Cabergoline decreases alcohol drinking and seeking behaviors via glial cell line-derived neurotrophic factor. Biol Psychiatry.

[CR35] Carnicella S, Ahmadiantehrani S, Janak PH, Ron D (2009). GDNF is an endogenous negative regulator of ethanol-mediated reward and of ethanol consumption after a period of abstinence. Alcohol Clin Exp Res.

[CR36] Carnicella S, Amamoto R, Ron D (2009). Excessive alcohol consumption is blocked by glial cell line-derived neurotrophic factor. Alcohol.

[CR37] Carnicella S, He DY, Yowell QV, Glick SD, Ron D (2010). Noribogaine, but not 18-MC, exhibits similar actions as ibogaine on GDNF expression and ethanol self-administration. Addict Biol.

[CR38] Carnicella S, Kharazia V, Jeanblanc J, Janak PH, Ron D (2008). GDNF is a fast-acting potent inhibitor of alcohol consumption and relapse. Proc Natl Acad Sci U S A.

[CR39] Carnicella S, Ron D, Barak S (2014). Intermittent ethanol access schedule in rats as a preclinical model of alcohol abuse. Alcohol.

[CR40] Castren E (2004). Neurotrophins as mediators of drug effects on mood, addiction, and neuroprotection. Mol Neurobiol.

[CR41] Chang PK, Yu L, Chen JC (2018). Dopamine D3 receptor blockade rescues hyper-dopamine activity-induced deficit in novel object recognition memory. Neuropharmacology.

[CR42] Chattopadhyaya B, Baho E, Huang ZJ, Schachner M, Di Cristo G (2013). Neural cell adhesion molecule-mediated Fyn activation promotes GABAergic synapse maturation in postnatal mouse cortex. J Neurosci.

[CR43] Chen ZY, Patel PD, Sant G, Meng CX, Teng KK, Hempstead BL, Lee FS (2004). Variant brain-derived neurotrophic factor (BDNF) (Met66) alters the intracellular trafficking and activity-dependent secretion of wild-type BDNF in neurosecretory cells and cortical neurons. J Neurosci.

[CR44] Chiarini F, Evangelisti C, McCubrey JA, Martelli AM (2015). Current treatment strategies for inhibiting mTOR in cancer. Trends Pharmacol Sci.

[CR45] Colzato LS, Van der Does AJ, Kouwenhoven C, Elzinga BM, Hommel B (2011). BDNF Val66Met polymorphism is associated with higher anticipatory cortisol stress response, anxiety, and alcohol consumption in healthy adults. Psychoneuroendocrinology.

[CR46] Costa-Mattioli M, Monteggia LM (2013). mTOR complexes in neurodevelopmental and neuropsychiatric disorders. Nat Neurosci.

[CR47] Costa-Mattioli M, Sossin WS, Klann E, Sonenberg N (2009). Translational control of long-lasting synaptic plasticity and memory. Neuron.

[CR48] Coultrap SJ, Bayer KU (2012). CaMKII regulation in information processing and storage. Trends Neurosci.

[CR49] Dadalko OI, Siuta M, Poe A, Erreger K, Matthies HJ, Niswender K, Galli A (2015). mTORC2/rictor signaling disrupts dopamine-dependent behaviors via defects in striatal dopamine neurotransmission. J Neurosci.

[CR50] Darcq E, Hamida SB, Wu S, Phamluong K, Kharazia V, Xu J, Lombroso P, Ron D (2014). Inhibition of striatal-enriched tyrosine phosphatase 61 in the dorsomedial striatum is sufficient to increased ethanol consumption. J Neurochem.

[CR51] Darcq E, Morisot N, Phamluong K, Warnault V, Jeanblanc J, Longo FM, Massa SM, Ron D (2016). The neurotrophic factor receptor p75 in the rat dorsolateral striatum drives excessive alcohol drinking. J Neurosci.

[CR52] Darcq E, Warnault V, Phamluong K, Besserer GM, Liu F, Ron D (2015). MicroRNA-30a-5p in the prefrontal cortex controls the transition from moderate to excessive alcohol consumption. Mol Psychiatry.

[CR53] Dayas CV, Smith DW, Dunkley PR (2012). An emerging role for the mammalian target of rapamycin in “pathological” protein translation: relevance to cocaine addiction. Front Pharmacol.

[CR54] Dowling RJ, Topisirovic I, Fonseca BD, Sonenberg N (2010). Dissecting the role of mTOR: lessons from mTOR inhibitors. Biochim Biophys Acta.

[CR55] Duman RS, Li N (2012). A neurotrophic hypothesis of depression: role of synaptogenesis in the actions of NMDA receptor antagonists. Philos Trans R Soc Lond Ser B Biol Sci.

[CR56] Dunah AW, Sirianni AC, Fienberg AA, Bastia E, Schwarzschild MA, Standaert DG (2004). Dopamine D1-dependent trafficking of striatal N-methyl-D-aspartate glutamate receptors requires Fyn protein tyrosine kinase but not DARPP-32. Mol Pharmacol.

[CR57] Easton AC, Lucchesi W, Mizuno K, Fernandes C, Schumann G, Giese KP, Muller CP (2013). alphaCaMKII autophosphorylation controls the establishment of alcohol-induced conditioned place preference in mice. Behav Brain Res.

[CR58] Egan MF, Kojima M, Callicott JH, Goldberg TE, Kolachana BS, Bertolino A, Zaitsev E, Gold B, Goldman D, Dean M, Lu B, Weinberger DR (2003). The BDNF val66met polymorphism affects activity-dependent secretion of BDNF and human memory and hippocampal function. Cell.

[CR59] Engen JR, Wales TE, Hochrein JM, Meyn MA, Banu Ozkan S, Bahar I, Smithgall TE (2008). Structure and dynamic regulation of Src-family kinases. Cell Mol Life Sci.

[CR60] Fernandez G, Lew B, Vedder L, DSavage L (2017). Chronic intermittent ethanol exposure leads to alterations in brain-derived neurotrophic factor within the frontal cortex and impaired behavioral flexibility in both adolescent and adult rats. Neuroscience.

[CR61] Friesland A, Weng Z, Duenas M, Massa SM, Longo FM, Lu Q (2014). Amelioration of cisplatin-induced experimental peripheral neuropathy by a small molecule targeting p75 NTR. Neurotoxicology.

[CR62] Gafford GM, Parsons RG, Helmstetter FJ (2011). Consolidation and reconsolidation of contextual fear memory requires mammalian target of rapamycin-dependent translation in the dorsal hippocampus. Neuroscience.

[CR63] Garcia-Marchena N, Silva-Pena D, Martin-Velasco AI, Villanua MA, Araos P, Pedraz M, Maza-Quiroga R, Romero-Sanchiz P, Rubio G, Castilla-Ortega E, Suarez J, Rodriguez de Fonseca F, Serrano A, Pavon FJ (2017). Decreased plasma concentrations of BDNF and IGF-1 in abstinent patients with alcohol use disorders. PLoS One.

[CR64] Gibb SL, Hamida SB, Lanfranco MF, Ron D (2011). Ethanol-induced increase in Fyn kinase activity in the dorsomedial striatum is associated with subcellular redistribution of protein tyrosine phosphatase alpha. J Neurochem.

[CR65] Glover EM, Ressler KJ, Davis M (2010). Differing effects of systemically administered rapamycin on consolidation and reconsolidation of context vs. cued fear memories. Learn Mem.

[CR66] Gobert D, Topolnik L, Azzi M, Huang L, Badeaux F, Desgroseillers L, Sossin WS, Lacaille JC (2008). Forskolin induction of late-LTP and up-regulation of 5' TOP mRNAs translation via mTOR, ERK, and PI3K in hippocampal pyramidal cells. J Neurochem.

[CR67] Goebel-Goody SM, Wilson-Wallis ED, Royston S, Tagliatela SM, Naegele JR, Lombroso PJ (2012). Genetic manipulation of STEP reverses behavioral abnormalities in a fragile X syndrome mouse model. Genes Brain Behav.

[CR68] Goodwani S, Saternos H, Alasmari F, Sari Y (2017). Metabotropic and ionotropic glutamate receptors as potential targets for the treatment of alcohol use disorder. Neurosci Biobehav Rev.

[CR69] Grant B, Chou S, Saha T, Pickering R, Kerridge B, Ruan W, Huang B, Jung J, Zhang H, Fan A, Hasin D (2017). Prevalence of 12-month alcohol use, high-risk drinking, and DSM-IV alcohol use disorder in the United States, 2001-2002 to 2012-2013: results from the National Epidemiologic Survey on alcohol and related conditions. JAMA Psychiatry.

[CR70] Grant SG, O'Dell TJ, Karl KA, Stein PL, Soriano P, Kandel ER (1992). Impaired long-term potentiation, spatial learning, and hippocampal development in fyn mutant mice. Science.

[CR71] Han J, Pollak J, Yang T, Siddiqui MR, Doyle KP, Taravosh-Lahn K, Cekanaviciute E, Han A, Goodman JZ, Jones B, Jing D, Massa SM, Longo FM, Buckwalter MS (2012). Delayed administration of a small molecule tropomyosin-related kinase B ligand promotes recovery after hypoxic-ischemic stroke. Stroke.

[CR72] Han S, Yang BZ, Kranzler HR, Liu X, Zhao H, Farrer LA, Boerwinkle E, Potash JB, Gelernter J (2013). Integrating GWASs and human protein interaction networks identifies a gene subnetwork underlying alcohol dependence. Am J Hum Genet.

[CR73] He DY, McGough NN, Ravindranathan A, Jeanblanc J, Logrip ML, Phamluong K, Janak PH, Ron D (2005). Glial cell line-derived neurotrophic factor mediates the desirable actions of the anti-addiction drug ibogaine against alcohol consumption. J Neurosci.

[CR74] He DY, Ron D (2006). Autoregulation of glial cell line-derived neurotrophic factor expression: implications for the long-lasting actions of the anti-addiction drug, Ibogaine. FASEB J.

[CR75] He DY, Ron D (2008). Glial cell line-derived neurotrophic factor reverses ethanol-mediated increases in tyrosine hydroxylase immunoreactivity via altering the activity of heat shock protein 90. J Biol Chem.

[CR76] Heberlein A, Muschler M, Wilhelm J, Frieling H, Lenz B, Groschl M, Kornhuber J, Bleich S, Hillemacher T (2010). BDNF and GDNF serum levels in alcohol-dependent patients during withdrawal. Prog Neuro-Psychopharmacol Biol Psychiatry.

[CR77] Heilig M, Barbier E, Johnstone AL, Tapocik J, Meinhardt MW, Pfarr S, Wahlestedt C, Sommer WH (2017). Reprogramming of mPFC transcriptome and function in alcohol dependence. Genes Brain Behav.

[CR78] Hensler JG, Ladenheim EE, Lyons WE (2003). Ethanol consumption and serotonin-1A (5-HT1A) receptor function in heterozygous BDNF (+/−) mice. J Neurochem.

[CR79] Hildebrand ME, Xu J, Dedek A, Li Y, Sengar AS, Beggs S, Lombroso PJ, Salter MW (2016). Potentiation of synaptic GluN2B NMDAR currents by Fyn kinase is gated through BDNF-mediated disinhibition in spinal pain processing. Cell Rep.

[CR80] Hoeffer CA, Klann E (2010). mTOR signaling: at the crossroads of plasticity, memory and disease. Trends Neurosci.

[CR81] Hoeffer CA, Tang W, Wong H, Santillan A, Patterson RJ, Martinez LA, Tejada-Simon MV, Paylor R, Hamilton SL, Klann E (2008). Removal of FKBP12 enhances mTOR-Raptor interactions, LTP, memory, and perseverative/repetitive behavior. Neuron.

[CR82] Huang EJ, Reichardt LF (2003). Trk receptors: roles in neuronal signal transduction. Annu Rev Biochem.

[CR83] Huang W, Zhu PJ, Zhang S, Zhou H, Stoica L, Galiano M, Krnjevic K, Roman G, Costa-Mattioli M (2013). mTORC2 controls actin polymerization required for consolidation of long-term memory. Nat Neurosci.

[CR84] Hwa L, Besheer J, Kash T (2017). Glutamate plasticity woven through the progression to alcohol use disorder: a multi-circuit perspective. F1000Res.

[CR85] Hyman SE, Malenka RC, Nestler EJ (2006). Neural mechanisms of addiction: the role of reward-related learning and memory. Annu Rev Neurosci.

[CR86] Ibanez CF, Andressoo JO (2017). Biology of GDNF and its receptors—relevance for disorders of the central nervous system. Neurobiol Dis.

[CR87] Ilagan E, Manning BD (2016). Emerging role of mTOR in the response to cancer therapeutics. Trends Cancer.

[CR88] Ishiguro H, Saito T, Shibuya H, Toru M, Arinami T (2000). Mutation and association analysis of the Fyn kinase gene with alcoholism and schizophrenia. Am J Med Genet.

[CR89] Jeanblanc J, Coune F, Botia B, Naassila M (2014). Brain-derived neurotrophic factor mediates the suppression of alcohol self-administration by memantine. Addict Biol.

[CR90] Jeanblanc J, He DY, Carnicella S, Kharazia V, Janak PH, Ron D (2009). Endogenous BDNF in the dorsolateral striatum gates alcohol drinking. J Neurosci.

[CR91] Jeanblanc J, He DY, McGough NN, Logrip ML, Phamluong K, Janak PH, Ron D (2006). The dopamine D3 receptor is part of a homeostatic pathway regulating ethanol consumption. J Neurosci.

[CR92] Jeanblanc J, Logrip ML, Janak PH, Ron D (2013). BDNF-mediated regulation of ethanol consumption requires the activation of the MAP kinase pathway and protein synthesis. Eur J Neurosci.

[CR93] Jernigan CS, Goswami DB, Austin MC, Iyo AH, Chandran A, Stockmeier CA, Karolewicz B (2011). The mTOR signaling pathway in the prefrontal cortex is compromised in major depressive disorder. Prog Neuro-Psychopharmacol Biol Psychiatry.

[CR94] Jobim PF, Pedroso TR, Werenicz A, Christoff RR, Maurmann N, Reolon GK, Schroder N, Roesler R (2012). Impairment of object recognition memory by rapamycin inhibition of mTOR in the amygdala or hippocampus around the time of learning or reactivation. Behav Brain Res.

[CR95] Khaled MA, Pushparaj A, Di Ciano P, Diaz J, Le Foll B (2014). Dopamine D3 receptors in the basolateral amygdala and the lateral habenula modulate cue-induced reinstatement of nicotine seeking. Neuropsychopharmacology.

[CR96] Knowles JK, Simmons DA, Nguyen TV, Vander Griend L, Xie Y, Zhang H, Yang T, Pollak J, Chang T, Arancio O, Buckwalter MS, Wyss-Coray T, Massa SM, Longo FM (2013). Small molecule p75NTR ligand prevents cognitive deficits and neurite degeneration in an Alzheimer’s mouse model. Neurobiol Aging.

[CR97] Kojima N, Wang J, Mansuy IM, Grant SG, Mayford M, Kandel ER (1997). Rescuing impairment of long-term potentiation in Fyn-deficient mice by introducing Fyn transgene. Proc Natl Acad Sci U S A.

[CR98] Koob GF, Volkow ND (2010). Neurocircuitry of addiction. Neuropsychopharmacology.

[CR99] Kraemer BR, Snow JP, Vollbrecht P, Pathak A, Valentine WM, Deutch AY, Carter BD (2014). A role for the p75 neurotrophin receptor in axonal degeneration and apoptosis induced by oxidative stress. J Biol Chem.

[CR100] Kraemer BR, Yoon SO, Carter BD (2014). The biological functions and signaling mechanisms of the p75 neurotrophin receptor. Handb Exp Pharmacol.

[CR101] Krauss GL, Edwards HB, Lin B (2012). Lacosamide for the treatment of epilepsy. Ann Med.

[CR102] Krishnan-Sarin S, O'Malley SS, Franco N, Cavallo DA, Morean M, Shi J, Pittman B, Krystal JH (2015). N-Methyl-D-aspartate receptor antagonism has differential effects on alcohol craving and drinking in heavy drinkers. Alcohol Clin Exp Res.

[CR103] Krupitsky EM, Neznanova O, Masalov D, Burakov AM, Didenko T, Romanova T, Tsoy M, Bespalov A, Slavina TY, Grinenko AA, Petrakis IL, Pittman B, Gueorguieva R, Zvartau EE, Krystal JH (2007). Effect of memantine on cue-induced alcohol craving in recovering alcohol-dependent patients. Am J Psychiatry.

[CR104] Krupitsky EM, Rudenko AA, Burakov AM, Slavina TY, Grinenko AA, Pittman B, Gueorguieva R, Petrakis IL, Zvartau EE, Krystal JH (2007). Antiglutamatergic strategies for ethanol detoxification: comparison with placebo and diazepam. Alcohol Clin Exp Res.

[CR105] Laguesse S, Morisot N, Phamluong K, Ron D (2016) Region specific activation of the AKT and mTORC1 pathway in response to excessive alcohol intake in rodents. Addict Biol 10.1111/adb.12464 PMC5398951 27766766

[CR106] Laguesse S, Morisot N, Phamluong K, Sakhai SA, Ron D (2018) mTORC2 in the dorsomedial striatum of mice contributes to alcohol-dependent F-actin polymerization, structural modifications and consumption. Neuropsychopharmacology: Accepted 10.1038/s41386-018-0012-1 PMC5983552 29497165

[CR107] Laguesse S, Morisot N, Shin JH, Liu F, Adrover MF, Sakhai SA, Lopez MF, Phamluong K, Griffin WC, Becker HC, Bender KJ, Alvarez VA, Ron D (2017). Prosapip1-dependent synaptic adaptations in the nucleus Accumbens drive alcohol intake, seeking, and reward. Neuron.

[CR108] Legastelois R, Darcq E, Wegner SA, Lombroso PJ, Ron D (2015). Striatal-enriched protein tyrosine phosphatase controls responses to aversive stimuli: implication for ethanol drinking. PLoS One.

[CR109] Li J, Kim SG, Blenis J (2014). Rapamycin: one drug, many effects. Cell Metab.

[CR110] Li TK, Lumeng L, McBride WJ, Murphy JM (1987). Rodent lines selected for factors affecting alcohol consumption. Alcohol Alcohol Suppl.

[CR111] Liao L, Pilotte J, Xu T, Wong CC, Edelman GM, Vanderklish P, Yates JR (2007). BDNF induces widespread changes in synaptic protein content and up-regulates components of the translation machinery: an analysis using high-throughput proteomics. J Proteome Res.

[CR112] Lin J, Liu L, Wen Q, Zheng C, Gao Y, Peng S, Tan Y, Li Y (2014). Rapamycin prevents drug seeking via disrupting reconsolidation of reward memory in rats. Int J Neuropsychopharmacol.

[CR113] Lin LF, Doherty DH, Lile JD, Bektesh S, Collins F (1993). GDNF: a glial cell line-derived neurotrophic factor for midbrain dopaminergic neurons. Science.

[CR114] Lipton JO, Sahin M (2014). The neurology of mTOR. Neuron.

[CR115] Liu F, Laguesse S, Legastelois R, Morisot N, Ben Hamida S, Ron D (2017). mTORC1-dependent translation of collapsin response mediator protein-2 drives neuroadaptations underlying excessive alcohol-drinking behaviors. Mol Psychiatry.

[CR116] Logrip ML, Barak S, Warnault V, Ron D (2015). Corticostriatal BDNF and alcohol addiction. Brain Res.

[CR117] Logrip ML, Janak PH, Ron D (2008). Dynorphin is a downstream effector of striatal BDNF regulation of ethanol intake. FASEB J.

[CR118] Logrip ML, Janak PH, Ron D (2009). Blockade of ethanol reward by the kappa opioid receptor agonist U50,488H. Alcohol.

[CR119] Logrip ML, Janak PH, Ron D (2009). Escalating ethanol intake is associated with altered corticostriatal BDNF expression. J Neurochem.

[CR120] Longo FM, Massa SM (2013). Small-molecule modulation of neurotrophin receptors: a strategy for the treatment of neurological disease. Nat Rev Drug Discov.

[CR121] Lopez MF, Becker HC (2014). Operant ethanol self-administration in ethanol dependent mice. Alcohol.

[CR122] Ma XM, Blenis J (2009). Molecular mechanisms of mTOR-mediated translational control. Nat Rev Mol Cell Biol.

[CR123] Mac Callum PE, Hebert M, Adamec RE, Blundell J (2014). Systemic inhibition of mTOR kinase via rapamycin disrupts consolidation and reconsolidation of auditory fear memory. Neurobiol Learn Mem.

[CR124] Malenka RC, Nicoll RA (1993). NMDA-receptor-dependent synaptic plasticity: multiple forms and mechanisms. Trends Neurosci.

[CR125] Malinow R, Malenka RC (2002). AMPA receptor trafficking and synaptic plasticity. Annu Rev Neurosci.

[CR126] Massa SM, Yang T, Xie Y, Shi J, Bilgen M, Joyce JN, Nehama D, Rajadas J, Longo FM (2010). Small molecule BDNF mimetics activate TrkB signaling and prevent neuronal degeneration in rodents. J Clin Invest.

[CR127] McGough NN, He DY, Logrip ML, Jeanblanc J, Phamluong K, Luong K, Kharazia V, Janak PH, Ron D (2004). RACK1 and brain-derived neurotrophic factor: a homeostatic pathway that regulates alcohol addiction. J Neurosci.

[CR128] Mendoza MC, Er EE, Blenis J (2011). The Ras-ERK and PI3K-mTOR pathways: cross-talk and compensation. Trends Biochem Sci.

[CR129] Meyers JL, Salling MC, Almli LM, Ratanatharathorn A, Uddin M, Galea S, Wildman DE, Aiello AE, Bradley B, Ressler K, Koenen KC (2015). Frequency of alcohol consumption in humans; the role of metabotropic glutamate receptors and downstream signaling pathways. Transl Psychiatry.

[CR130] Molina JR, Foster NR, Reungwetwattana T, Nelson GD, Grainger AV, Steen PD, Stella PJ, Marks R, Wright J, Adjei AA (2014). A phase II trial of the Src-kinase inhibitor saracatinib after four cycles of chemotherapy for patients with extensive stage small cell lung cancer: NCCTG trial N-0621. Lung Cancer.

[CR131] Moonat S, Sakharkar AJ, Zhang H, Pandey SC (2011). The role of amygdaloid brain-derived neurotrophic factor, activity-regulated cytoskeleton-associated protein and dendritic spines in anxiety and alcoholism. Addict Biol.

[CR132] Moonat S, Sakharkar AJ, Zhang H, Tang L, Pandey SC (2013). Aberrant histone deacetylase2-mediated histone modifications and synaptic plasticity in the amygdala predisposes to anxiety and alcoholism. Biol Psychiatry.

[CR133] Morisot N, Novotny CJ, Shokat KM, Ron D (2017) A new generation of mTORC1 inhibitor attenuates alcohol intake and reward in mice. Addict Biol 10.1111/adb.12528 28681511

[CR134] Morisot N, Ron D (2017). Alcohol-dependent molecular adaptations of the NMDA receptor system. Genes Brain Behav.

[CR135] Myskiw JC, Rossato JI, Bevilaqua LR, Medina JH, Izquierdo I, Cammarota M (2008). On the participation of mTOR in recognition memory. Neurobiol Learn Mem.

[CR136] Nakazawa T, Komai S, Tezuka T, Hisatsune C, Umemori H, Semba K, Mishina M, Manabe T, Yamamoto T (2001). Characterization of Fyn-mediated tyrosine phosphorylation sites on GluR epsilon 2 (NR2B) subunit of the N-methyl-D-aspartate receptor. J Biol Chem.

[CR137] Natividad LA, Steinman MQ, Laredo SA, Irimia C, Polis IY, Lintz R, Buczynski MW, Martin-Fardon R, Roberto M, Parsons LH (2017) Phosphorylation of calcium/calmodulin-dependent protein kinase II in the rat dorsal medial prefrontal cortex is associated with alcohol-induced cognitive inflexibility. Addict Biol 10.1111/adb.12568 PMC5862723 28940879

[CR138] Neasta J, Barak S, Hamida SB, Ron D (2014). mTOR complex 1: a key player in neuroadaptations induced by drugs of abuse. J Neurochem.

[CR139] Neasta J, Ben Hamida S, Yowell Q, Carnicella S, Ron D (2010). Role for mammalian target of rapamycin complex 1 signaling in neuroadaptations underlying alcohol-related disorders. Proc Natl Acad Sci U S A.

[CR140] Neasta J, Ben Hamida S, Yowell QV, Carnicella S, Ron D (2011). AKT signaling pathway in the nucleus accumbens mediates excessive alcohol drinking behaviors. Biol Psychiatry.

[CR141] Nees F, Witt SH, Dinu-Biringer R, Lourdusamy A, Tzschoppe J, Vollstadt-Klein S, Millenet S, Bach C, Poustka L, Banaschewski T, Barker GJ, Bokde AL, Bromberg U, Buchel C, Conrod PJ, Frank J, Frouin V, Gallinat J, Garavan H, Gowland P, Heinz A, Ittermann B, Mann K, Martinot JL, Paus T, Pausova Z, Robbins TW, Smolka MN, Rietschel M, Schumann G, Flor H, consortium I (2015). BDNF Val66Met and reward-related brain function in adolescents: role for early alcohol consumption. Alcohol.

[CR142] Newman-Tancredi A, Cussac D, Audinot V, Nicolas JP, De Ceuninck F, Boutin JA, Millan MJ (2002). Differential actions of antiparkinson agents at multiple classes of monoaminergic receptor. II. Agonist and antagonist properties at subtypes of dopamine D(2)-like receptor and alpha(1)/alpha(2)-adrenoceptor. J Pharmacol Exp Ther.

[CR143] Noller GE, Frampton CM, Yazar-Klosinski B (2017) Ibogaine treatment outcomes for opioid dependence from a twelve-month follow-up observational study. Am J Drug Alcohol Abuse:1–10 10.1080/00952990.2017.1310218 28402682

[CR144] Nubukpo P, Ramoz N, Girard M, Malauzat D, Gorwood P (2017). Determinants of blood brain-derived neurotrophic factor blood levels in patients with alcohol use disorder. Alcohol Clin Exp Res.

[CR145] Nygaard HB, Wagner AF, Bowen GS, Good SP, MacAvoy MG, Strittmatter KA, Kaufman AC, Rosenberg BJ, Sekine-Konno T, Varma P, Chen K, Koleske AJ, Reiman EM, Strittmatter SM, van Dyck CH (2015). A phase Ib multiple ascending dose study of the safety, tolerability, and central nervous system availability of AZD0530 (saracatinib) in Alzheimer’s disease. Alzheimers Res Ther.

[CR146] Ogden KK, Traynelis SF (2011). New advances in NMDA receptor pharmacology. Trends Pharmacol Sci.

[CR147] Oh WJ, Jacinto E (2011). mTOR complex 2 signaling and functions. Cell Cycle.

[CR148] Ohnishi H, Murata Y, Okazawa H, Matozaki T (2011). Src family kinases: modulators of neurotransmitter receptor function and behavior. Trends Neurosci.

[CR149] Orru A, Caffino L, Moro F, Cassina C, Giannotti G, Di Clemente A, Fumagalli F, Cervo L (2016). Contingent and non-contingent recreational-like exposure to ethanol alters BDNF expression and signaling in the cortico-accumbal network differently. Psychopharmacology.

[CR150] Pandey SC, Zhang H, Ugale R, Prakash A, Xu T, Misra K (2008). Effector immediate-early gene arc in the amygdala plays a critical role in alcoholism. J Neurosci.

[CR151] Panja D, Bramham CR (2014). BDNF mechanisms in late LTP formation: a synthesis and breakdown. Neuropharmacology.

[CR152] Park H, Poo MM (2013). Neurotrophin regulation of neural circuit development and function. Nat Rev Neurosci.

[CR153] Pascual A, Hidalgo-Figueroa M, Piruat JI, Pintado CO, Gomez-Diaz R, Lopez-Barneo J (2008). Absolute requirement of GDNF for adult catecholaminergic neuron survival. Nat Neurosci.

[CR154] Pastor IJ, Laso FJ, Ines S, Marcos M, Gonzalez-Sarmiento R (2009). Genetic association between -93A/G polymorphism in the Fyn kinase gene and alcohol dependence in Spanish men. Eur Psychiatry.

[CR155] Phamluong K, Darcq E, Wu S, Sakhai SA, Ron D (2017). Fyn signaling is compartmentalized to dopamine D1 receptor expressing neurons in the dorsal medial striatum. Front Mol Neurosci.

[CR156] Pochon NA, Menoud A, Tseng JL, Zurn AD, Aebischer P (1997). Neuronal GDNF expression in the adult rat nervous system identified by in situ hybridization. Eur J Neurosci.

[CR157] Ponniah S, Wang DZ, Lim KL, Pallen CJ (1999). Targeted disruption of the tyrosine phosphatase PTPalpha leads to constitutive downregulation of the kinases Src and Fyn. Curr Biol.

[CR158] Prybylowski K, Chang K, Sans N, Kan L, Vicini S, Wenthold RJ (2005). The synaptic localization of NR2B-containing NMDA receptors is controlled by interactions with PDZ proteins and AP-2. Neuron.

[CR159] Quach TT, Honnorat J, Kolattukudy PE, Khanna R, Duchemin AM (2015) CRMPs: critical molecules for neurite morphogenesis and neuropsychiatric diseases. Mol Psychiatry 20:1037–45 10.1038/mp.2015.77 26077693

[CR160] Rodrik-Outmezguine VS, Okaniwa M, Yao Z, Novotny CJ, McWhirter C, Banaji A, Won H, Wong W, Berger M, de Stanchina E, Barratt DG, Cosulich S, Klinowska T, Rosen N, Shokat KM (2016). Overcoming mTOR resistance mutations with a new-generation mTOR inhibitor. Nature.

[CR161] Ron D (2004). Signaling cascades regulating NMDA receptor sensitivity to ethanol. Neuroscientist.

[CR162] Ron D, Barak S (2016). Molecular mechanisms underlying alcohol-drinking behaviours. Nat Rev Neurosci.

[CR163] Ron D, Wang J (2009) The NMDA receptor and alcohol addiction. In: Van Dongen AM (ed) Biology of the NMDA Receptor (Frontiers in Neuroscience), Boca Raton (FL)

[CR164] Salling MC, Faccidomo SP, Li C, Psilos K, Galunas C, Spanos M, Agoglia AE, Kash TL, Hodge CW (2016). Moderate alcohol drinking and the amygdala proteome: identification and validation of calcium/calmodulin dependent kinase II and AMPA receptor activity as novel molecular mechanisms of the positive reinforcing effects of alcohol. Biol Psychiatry.

[CR165] Saxton RA, Sabatini DM (2017). mTOR signaling in growth, metabolism, and disease. Cell.

[CR166] Schmid DA, Yang T, Ogier M, Adams I, Mirakhur Y, Wang Q, Massa SM, Longo FM, Katz DM (2012). A TrkB small molecule partial agonist rescues TrkB phosphorylation deficits and improves respiratory function in a mouse model of Rett syndrome. J Neurosci.

[CR167] Schumann G, Rujescu D, Kissling C, Soyka M, Dahmen N, Preuss UW, Wieman S, Depner M, Wellek S, Lascorz J, Bondy B, Giegling I, Anghelescu I, Cowen MS, Poustka A, Spanagel R, Mann K, Henn FA, Szegedi A (2003). Analysis of genetic variations of protein tyrosine kinase fyn and their association with alcohol dependence in two independent cohorts. Biol Psychiatry.

[CR168] Schwarze SR, Ho A, Vocero-Akbani A, Dowdy SF (1999). In vivo protein transduction: delivery of a biologically active protein into the mouse. Science.

[CR169] Sheppard SG (1994). A preliminary investigation of ibogaine: case reports and recommendations for further study. J Subst Abus Treat.

[CR170] Shoptaw S, Watson DW, Reiber C, Rawson RA, Montgomery MA, Majewska MD, Ling W (2005). Randomized controlled pilot trial of cabergoline, hydergine and levodopa/carbidopa: Los Angeles Cocaine Rapid Efficacy Screening Trial (CREST). Addiction.

[CR171] Simmons DA, Belichenko NP, Ford EC, Semaan S, Monbureau M, Aiyaswamy S, Holman CM, Condon C, Shamloo M, Massa SM, Longo FM (2016). A small molecule p75NTR ligand normalizes signalling and reduces Huntington’s disease phenotypes in R6/2 and BACHD mice. Hum Mol Genet.

[CR172] Simmons DA, Belichenko NP, Yang T, Condon C, Monbureau M, Shamloo M, Jing D, Massa SM, Longo FM (2013). A small molecule TrkB ligand reduces motor impairment and neuropathology in R6/2 and BACHD mouse models of Huntington’s disease. J Neurosci.

[CR173] Simmons DA, Knowles JK, Belichenko NP, Banerjee G, Finkle C, Massa SM, Longo FM (2014). A small molecule p75NTR ligand, LM11A-31, reverses cholinergic neurite dystrophy in Alzheimer’s disease mouse models with mid- to late-stage disease progression. PLoS One.

[CR174] Slipczuk L, Bekinschtein P, Katche C, Cammarota M, Izquierdo I, Medina JH (2009). BDNF activates mTOR to regulate GluR1 expression required for memory formation. PLoS One.

[CR175] Smith ML, Lopez MF, Archer KJ, Wolen AR, Becker HC, Miles MF (2016). Time-course analysis of brain regional expression network responses to chronic intermittent ethanol and withdrawal: implications for mechanisms underlying excessive ethanol consumption. PLoS One.

[CR176] Sridhar D (2012). Health policy: regulate alcohol for global health. Nature.

[CR177] Stoica L, Zhu PJ, Huang W, Zhou H, Kozma SC, Costa-Mattioli M (2011). Selective pharmacogenetic inhibition of mammalian target of rapamycin complex I (mTORC1) blocks long-term synaptic plasticity and memory storage. Proc Natl Acad Sci U S A.

[CR178] Sui L, Wang J, Li BM (2008). Role of the phosphoinositide 3-kinase-Akt-mammalian target of the rapamycin signaling pathway in long-term potentiation and trace fear conditioning memory in rat medial prefrontal cortex. Learn Mem.

[CR179] Tang SJ, Reis G, Kang H, Gingras AC, Sonenberg N, Schuman EM (2002). A rapamycin-sensitive signaling pathway contributes to long-term synaptic plasticity in the hippocampus. Proc Natl Acad Sci U S A.

[CR180] Tapocik JD, Barbier E, Flanigan M, Solomon M, Pincus A, Pilling A, Sun H, Schank JR, King C, Heilig M (2014). microRNA-206 in rat medial prefrontal cortex regulates BDNF expression and alcohol drinking. J Neurosci.

[CR181] Tep C, Lim TH, Ko PO, Getahun S, Ryu JC, Goettl VM, Massa SM, Basso M, Longo FM, Yoon SO (2013). Oral administration of a small molecule targeted to block proNGF binding to p75 promotes myelin sparing and functional recovery after spinal cord injury. J Neurosci.

[CR182] Thomanetz V, Angliker N, Cloetta D, Lustenberger RM, Schweighauser M, Oliveri F, Suzuki N, Ruegg MA (2013). Ablation of the mTORC2 component rictor in brain or Purkinje cells affects size and neuron morphology. J Cell Biol.

[CR183] Thoreen CC, Chantranupong L, Keys HR, Wang T, Gray NS, Sabatini DM (2012). A unifying model for mTORC1-mediated regulation of mRNA translation. Nature.

[CR184] Tomac A, Widenfalk J, Lin LF, Kohno T, Ebendal T, Hoffer BJ, Olson L (1995). Retrograde axonal transport of glial cell line-derived neurotrophic factor in the adult nigrostriatal system suggests a trophic role in the adult. Proc Natl Acad Sci U S A.

[CR185] Tong M, Jiang Y (2015). FK506-binding proteins and their diverse functions. Curr Mol Pharmacol.

[CR186] Trepanier CH, Jackson MF, MacDonald JF (2012). Regulation of NMDA receptors by the tyrosine kinase Fyn. FEBS J.

[CR187] Trupp M, Belluardo N, Funakoshi H, Ibanez CF (1997). Complementary and overlapping expression of glial cell line-derived neurotrophic factor (GDNF), c-ret proto-oncogene, and GDNF receptor-alpha indicates multiple mechanisms of trophic actions in the adult rat CNS. J Neurosci.

[CR188] Vacaresse N, Moller B, Danielsen EM, Okada M, Sap J (2008). Activation of c-Src and Fyn kinases by protein-tyrosine phosphatase RPTPalpha is substrate-specific and compatible with lipid raft localization. J Biol Chem.

[CR189] Vengeliene V, Bilbao A, Spanagel R (2014). The alcohol deprivation effect model for studying relapse behavior: a comparison between rats and mice. Alcohol.

[CR190] Wackernah RC, Minnick MJ, Clapp P (2014). Alcohol use disorder: pathophysiology, effects, and pharmacologic options for treatment. Subst Abuse Rehabil.

[CR191] Wang J, Ben Hamida S, Darcq E, Zhu W, Gibb SL, Lanfranco MF, Carnicella S, Ron D (2012). Ethanol-mediated facilitation of AMPA receptor function in the dorsomedial striatum: implications for alcohol drinking behavior. J Neurosci.

[CR192] Wang J, Carnicella S, Phamluong K, Jeanblanc J, Ronesi JA, Chaudhri N, Janak PH, Lovinger DM, Ron D (2007). Ethanol induces long-term facilitation of NR2B-NMDA receptor activity in the dorsal striatum: implications for alcohol drinking behavior. J Neurosci.

[CR193] Wang J, Lanfranco MF, Gibb SL, Yowell QV, Carnicella S, Ron D (2010). Long-lasting adaptations of the NR2B-containing NMDA receptors in the dorsomedial striatum play a crucial role in alcohol consumption and relapse. J Neurosci.

[CR194] Wang J, Carnicella S, Ahmadiantehrani S, He DY, Barak S, Kharazia V, Ben Hamida S, Zapata A, Shippenberg TS, Ron D (2010b) Nucleus accumbens-derived glial cell line-derived neurotrophic factor is a retrograde enhancer of dopaminergic tone in the mesocorticolimbic system. J Neurosci 30:14502–12 10.1523/JNEUROSCI.3909-10.2010 PMC2981598 20980608

[CR195] Warnault V, Darcq E, Morisot N, Phamluong K, Wilbrecht L, Massa SM, Longo FM, Ron D (2016). The BDNF valine 68 to methionine polymorphism increases compulsive alcohol drinking in mice that is reversed by tropomyosin receptor kinase B activation. Biol Psychiatry.

[CR196] Webster J, Piscitelli G, Polli A, Ferrari CI, Ismail I, Scanlon MF (1994). A comparison of cabergoline and bromocriptine in the treatment of hyperprolactinemic amenorrhea. Cabergoline Comparative Study Group. N Engl J Med.

[CR197] Wendholt D, Spilker C, Schmitt A, Dolnik A, Smalla KH, Proepper C, Bockmann J, Sobue K, Gundelfinger ED, Kreutz MR, Boeckers TM (2006). ProSAP-interacting protein 1 (ProSAPiP1), a novel protein of the postsynaptic density that links the spine-associated rap-gap (SPAR) to the scaffolding protein ProSAP2/Shank3. J Biol Chem.

[CR198] Whiteford HA, Degenhardt L, Rehm J, Baxter AJ, Ferrari AJ, Erskine HE, Charlson FJ, Norman RE, Flaxman AD, Johns N, Burstein R, Murray CJ, Vos T (2013). Global burden of disease attributable to mental and substance use disorders: findings from the Global Burden of Disease Study 2010. Lancet.

[CR199] WHO (2014) World Health Statistics 2014. World Health Organization, pp 1–180

[CR200] Wilson SM, Khanna R (2015). Specific binding of lacosamide to collapsin response mediator protein 2 (CRMP2) and direct impairment of its canonical function: implications for the therapeutic potential of lacosamide. Mol Neurobiol.

[CR201] Wojnar M, Brower KJ, Strobbe S, Ilgen M, Matsumoto H, Nowosad I, Sliwerska E, Burmeister M (2009). Association between Val66Met brain-derived neurotrophic factor (BDNF) gene polymorphism and post-treatment relapse in alcohol dependence. Alcohol Clin Exp Res.

[CR202] Woo NH, Teng HK, Siao CJ, Chiaruttini C, Pang PT, Milner TA, Hempstead BL, Lu B (2005). Activation of p75NTR by proBDNF facilitates hippocampal long-term depression. Nat Neurosci.

[CR203] Yaka R, Phamluong K, Ron D (2003). Scaffolding of Fyn kinase to the NMDA receptor determines brain region sensitivity to ethanol. J Neurosci.

[CR204] Yaka R, Thornton C, Vagts AJ, Phamluong K, Bonci A, Ron D (2002). NMDA receptor function is regulated by the inhibitory scaffolding protein, RACK1. Proc Natl Acad Sci U S A.

[CR205] Yan QS, Feng MJ, Yan SE (2005). Different expression of brain-derived neurotrophic factor in the nucleus accumbens of alcohol-preferring (P) and -nonpreferring (NP) rats. Brain Res.

[CR206] Yang F, Feng L, Zheng F, Johnson SW, Du J, Shen L, Wu CP, Lu B (2001). GDNF acutely modulates excitability and A-type K(+) channels in midbrain dopaminergic neurons. Nat Neurosci.

[CR207] Yang T, Knowles JK, Lu Q, Zhang H, Arancio O, Moore LA, Chang T, Wang Q, Andreasson K, Rajadas J, Fuller GG, Xie Y, Massa SM, Longo FM (2008). Small molecule, non-peptide p75 ligands inhibit Abeta-induced neurodegeneration and synaptic impairment. PLoS One.

[CR208] Yip CK, Murata K, Walz T, Sabatini DM, Kang SA (2010). Structure of the human mTOR complex I and its implications for rapamycin inhibition. Mol Cell.

[CR209] Zagrebelsky M, Holz A, Dechant G, Barde YA, Bonhoeffer T, Korte M (2005). The p75 neurotrophin receptor negatively modulates dendrite complexity and spine density in hippocampal neurons. J Neurosci.

[CR210] Zanardini R, Fontana A, Pagano R, Mazzaro E, Bergamasco F, Romagnosi G, Gennarelli M, Bocchio-Chiavetto L (2011). Alterations of brain-derived neurotrophic factor serum levels in patients with alcohol dependence. Alcohol Clin Exp Res.

[CR211] Zinzalla V, Stracka D, Oppliger W, Hall MN (2011). Activation of mTORC2 by association with the ribosome. Cell.

[CR212] Zipori D, Sadot-Sogrin Y, Goltseker K, Even-Chen O, Rahamim N, Shaham O, Barak S (2017). Re-exposure to nicotine-associated context from adolescence enhances alcohol intake in adulthood. Sci Rep.

[CR213] Zoncu R, Efeyan A, Sabatini DM (2011). mTOR: from growth signal integration to cancer, diabetes and ageing. Nat Rev Mol Cell Biol.

